# *De novo* Assembly and Characterization of the Fruit Transcriptome of *Idesia polycarpa* Reveals Candidate Genes for Lipid Biosynthesis

**DOI:** 10.3389/fpls.2016.00801

**Published:** 2016-06-07

**Authors:** Rong-Jun Li, Xiang Gao, Lin-Mao Li, Xiu-Lin Liu, Zhou-Ya Wang, Shi-you Lü

**Affiliations:** ^1^Key Laboratory of Plant Germplasm Enhancement and Specialty Agriculture, Wuhan Botanical Garden, Chinese Academy of SciencesWuhan, China; ^2^Sino-Africa Joint Research Center, Chinese Academy of SciencesWuhan, China; ^3^University of Chinese Academy of SciencesBeijing, China

**Keywords:** *Idesia polycarpa*, transcriptome, seed, pericarp, lipid biosynthesis

## Abstract

*Idesia polycarpa*, is a valuable oilseed-producing tree of the Flacourtiaceae family that has the potential to fulfill edible oil production and is also a possible biofuel feedstock. The fruit is unique in that it contains both saturated and unsaturated lipids present in pericarp and seed, respectively. However, triglyceride synthesis and storage in tissues outside of the seeds has been poorly studied in previous researches. To gain insight into the unique properties of *I. polycarpa* fruit lipid synthesis, biochemical, and transcriptomic approaches were used to compare the lipid accumulation between pericarp and seed of the fruit. Lipid accumulation rates, final lipid content and composition were significantly different between two tissues. Furthermore, we described the annotated transcriptome assembly and differential gene expression analysis generated from the pericarp and seed tissues. The data allowed the identification of distinct candidate genes and reconstruction of lipid pathways, which may explain the differences of oil synthesis between the two tissues. The results may be useful for engineering alternative pathways for lipid production in non-seed or vegetative tissues.

## Introduction

Plant oils, mainly composed of triacylglycerols (TAGs), are an essential resource for human and animal food, the chemical industry and renewable energy (Durrett et al., 2008). It is predicted that demand for vegetable oils will be doubled by 2030, which can be met only by increasing the oil content in presently used oil crops or introducing new high-oil-yielding crops (Chapman and Ohlrogge, 2012). Much progress has been made in understanding how plants produce and accumulate oils. The specific enzymes involved in the metabolic pathway leading to TAGs stored in the oil bodies, as well as the pathway that supplies the precursors generated from imported sucrose, are well-known (Bates et al., 2013; Li-Beisson et al., 2013). However, this knowledge has mostly been achieved using typical oilseeds especially with *Arabidopsis* as a model plant. In these plants, the oil is mainly accumulating in the seed (Baud and Lepiniec, 2010). To enable a substantial increase in vegetable oil production, it is therefore important to also seek those plants with oil accumulation in other tissues (Xu and Shanklin, 2016).

Although seeds are by far the greatest current commercial sources of plant oils, abundant oil are capable from many other tissues. Oil accumulation clearly occurs in non-seed tissues in a number of plants. However, the ability of non-seed cells and tissues to accumulate TAG varies substantially. For example, lipids are not particularly abundant in leaf tissues, but are prevalent in some fruits (such as avocado, oil palm, olive), roots/tubers (such as cotton, nutsedge), floral tissues and even stems (Mongolian oil wood) of certain species (Durrett et al., 2008; Turesson et al., 2010; Xu and Shanklin, 2016). Even within the species that accumulate oil as a major seed storage reserve, substantial diversity is observed in TAG structure, rate of oil synthesis, level of accumulation, and whether oil is stored in the embryo or endosperm tissue (Baud and Lepiniec, 2010). Despite of extensive studies for more than 30 years, a number of molecular and biochemical factors associated with these variations among oilseeds remain poorly understood. To gain insight into conserved and diverse aspects of lipid metabolism across multiple species, it is useful to expand the genomic and transcriptomic resources available for non-model species to allow comparative analyses (Bates et al., 2013).

Oil palm is one of the most productive oil producing crops that can store up to 90% oil in its fruit mesocarp. Due to its economic importance, extensive research has focused on elucidating the underlying mechanisms and pathways influencing the efficient oil production machinery in the oil palm mesocarp tissue (Parveez et al., 2015). Recently, Bourgis et al. (2011) compared the differences of transcriptome and metabolome between oil palm and date palm during mesocarp development, in order to reveal the mechanisms that cause an extreme difference in carbon partitioning between them (the mesocarp of oil palm accumulates oil while the mesocarp of date palm accumulates sugars). Their results indicate that the synthesis of fatty acids and the supply of pyruvate in the plastid, rather than acyl assembly into TAGs, are the main factors for the accumulation of oil in the mesocarp of oil palm. Tranbarger et al. (2011) and Dussert et al. (2013) investigated the transcriptional basis of lipid accumulation in the mesocarp of oil palm. A transcript, homologous to *Arabidopsis* seed oil transcription factor *WRINKLED1* (*WRI1*), was identified to coordinate its transcript level with several fatty acids biosynthetic transcripts and high rates of lipid deposition, suggesting that the mesocarp homolog of *WRI1* is an important regulatory factor in oil biosynthesis.

Bayberry (*Myrica pensylvanica*) fruits synthesize an extremely thick and unusual layer of crystalline surface wax that accumulates up to 32% of dry fruit weight, the highest reported surface lipid accumulation in plants (Simpson and Ohlrogge, 2016). Recently, it was strikingly found that the surface wax is primarily composed of glycerolipids, notably triacylglycerol and diacylglycerol with saturated fatty acids. Being the only plant known to accumulate soluble glycerolipids as a major component of surface waxes, Bayberry represents a novel system for investigating neutral lipid biosynthesis and lipid secretion by vegetative plant cells (Simpson and Ohlrogge, 2016).

The accumulation of plant vegetative-oil presents an opportunity to create novel renewable approaches for expanded production of TAGs as a renewable and sustainable bioenergy source. Recently, much higher levels of oil accumulation in plant biomass were achieved using a combination of biotechnological approaches (Vanhercke et al., 2014). This accumulation of up to 15% TAG in *Nicotiana tabacum* leaves was achieved by the coordinated transgenic expression of *WRI1*, diacylglycerol acyltransferase (*DGAT1*) and oleosin genes. This breakthrough in leaf oil accumulation was ascribed to the synergistic increase in both fatty acid synthesis and oil synthesis (via *WRI1* and *DGAT1*, respectively) and the formation of stabilized oil bodies (via oleosin). Several reports on the use of metabolic engineering strategies to achieve vegetative-oil accumulation were recently reviewed by Xu and Shanklin (2016).

*Idesia polycarpa* is a dioecious tree of the Flacourtiaceae family. This tree is native to some Asian countries, including Korea, Japan, and China (Yang et al., 2009). Because of its adaptability and beautiful appearance, *I. polycarpa* is an ideal plant for gardeners in China. The oil from its fruit contains high quantities of unsaturated fatty acids especially linoleic acid, which is an ideal raw material for the edible oil and energy industry. In fact, the fruit of this plant has historically been used to prepare edible oil in China (Yang et al., 2009). Furthermore, the fruits have potential medicinal uses with a variety of compounds such as idesolide, which may be useful in combating obesity (Hwang et al., 2012). Recently, the feasibility of producing biodiesel from *I. polycarpa* fruit oil was also studied. The fuel properties of biodiesel obtained from *I. polycarpa* fruit oil are similar to the No. 0 light diesel fuel and most of the parameters comply with the specification limits established for biodiesel. Therefore, the *I. polycarpa* fruit oil can be potentially used as a raw feedstock for producing biodiesel on a commercial scale (Yang et al., 2009).

In this paper, our objective is to provide a basis to understand the molecular regulation and coordination of TAG biosynthesis during *I. polycarpa* fruits development, focusing on the comparison between the seed and pericarp. We studied oil spatial and temporal accumulation patterns, transcriptome sequencings, and differential gene expression profiles. We describe the unique characters of lipid metabolism in this species and provide insight into the transcriptional coordination in the seed and pericarp tissues. Finally, candidate genes responsible for the oil content and fatty acid compositions difference between seed and pericap were proposed. Our studies will serve as an important foundation to further explore the regulatory mechanism of *I. polycarpa* fruit oil accumulation, and may also provide a clue for researching the non-seed woody biodiesel plants.

## Materials and methods

### Plant materials

Plants used for this study were grown at Huanggang, Hubei province. Fruits from trees were handpicked, approximately at bi-weekly intervals, at 13 distinct stages of fruit development, referred to as days after pollination (DAP). A portion of the fruits was subjected to pericarp and seed separation with the aid of a scalpel. The isolated tissues were weighed and flash frozen in liquid nitrogen and stored at −80°C until further use. For final oil content determinations, fruits from 73 *I. polycarpa* accessions were collected at harvest time.

### Oil content determination

The fruits of *I. polycarpa* were dried at 60°C and crushed into powder. Two grams powder was put into a filter paper pack. Oil was extracted by Soxhlet extraction for 3 h. The solvent was petroleum ether. The oil content was then calculated from the formula: whole fruit oil content = (filter paper pack dry weight before extraction—filter paper pack dry weight after extraction)/fruit powder dry weight × 100%.

### Fatty acid composition determination

To determine the fatty acid compositions, 15~20 mg fruit powder was weighed and put into a glass tube. Then 1 ml of Hexane was added and vortexed for about 10 s. After that, 200 μl of KOH methanol solution and 20 μl of BHT solution (0.2% butylated hydroxy toluene in methanol) were added and mixed. Then the total oil was extracted by an ultrasonic cleaner for 2 min. Finally, the mixture was centrifuged at 12000 rpm for 10 min and the compounds of fatty acid methyl esters (FAMEs) in the upper organic phase were removed for gas chromatograph analysis (Agilent 7820A, CA) with a flame ionization detector (FID) on a DB-23 colume (0.25 mm × 30 m, 0.25 μm). The GC conditions were: column oven temperature 170°Cand flame ionization detector set at 280°C.

### Library preparation for transcriptome sequencing

Total RNAs were isolated from the seed and pericarp tissues from 60 DAP respectively, using Trizol reagent (Invitrogen, CA, USA) according to the manufacturer's protocol. The RNA quality was evaluated by electrophoresis through a 1% agarose gel, and the RNA concentration was determined by absorbance at 260 nm using a Nanodrop spectrophotometer (Nanodrop Technologies, USA). A total amount of 1.5 μg RNA per sample was used as input material for the RNA sample preparations. Sequencing libraries were generated using NEBNext® Ultra™ RNA Library Prep Kit for Illumina® (NEB, USA) following manufacturer's recommendations and index codes were added to attribute sequences to each sample. Briefly, mRNA was purified from total RNA using poly-T oligo-attached magnetic beads. Fragmentation was carried out using divalent cations under elevated temperature in NEBNext First Strand Synthesis Reaction Buffer (5X). First strand cDNA was synthesized using random hexamer primer and M-MuLV Reverse Transcriptase (RNase H-). Second strand cDNA synthesis was subsequently performed using DNA Polymerase I and RNase H. Remaining overhangs were converted into blunt ends via exonuclease/polymerase activities. After adenylation of 3′ ends of DNA fragments, NEBNext Adaptor with hairpin loop structure were ligated to prepare for hybridization. In order to select cDNA fragments of preferentially 150~200 bp in length, the library fragments were purified with AMPure XP system (Beckman Coulter, Beverly, USA). Then 3 μl USER Enzyme (NEB, USA) was used with size-selected, adaptor-ligated cDNA at 37°C for 15 min followed by 5 min at 95°C before PCR. Then PCR was performed with Phusion High-Fidelity DNA polymerase, Universal PCR primers and Index (X) Primer. At last, PCR products were purified (AMPure XP system) and library quality was assessed on the Agilent Bioanalyzer 2100 system. Sequencing was conducted at Beijing Novogene Biological Information Technology Co., Ltd., Beijing, China (http://www.novogene.com/) using the Illumina TruSeqTM RNA Sample Preparation Kit (Illumina, San Diego, CA, USA) following the manufacturer's recommendations. The clustering of the index-coded samples was performed on a cBot Cluster Generation System using TruSeq PE Cluster Kit v3-cBot-HS (Illumia) according to the manufacturer's instructions. After cluster generation, the library preparations were sequenced on an Illumina Hiseq platform and paired-end reads were generated.

### Transcriptome assembly

Raw data (raw reads) of fastq format were firstly processed through in-house perl scripts. In this step, clean data(clean reads) were obtained by removing reads containing adapter, reads containing ploy-N and low quality reads from raw data. At the same time, Q20, Q30, GC-content and sequence duplication level of the clean data were calculated. All the downstream analyses were based on clean data with high quality. The left files (read1 files) from all libraries/samples were pooled into one big left.fq file, and right files (read2 files) into one big right.fq file. Transcriptome assembly was accomplished based on the left.fq and right.fq using Trinity (Grabherr et al., 2011) with min_kmer_cov set to 25 by default and all other parameters set default.

### Gene functional annotation

Gene function was annotated based on the following databases: NR (NCBI non-redundant protein sequences); Nt (NCBI non-redundant nucleotide sequences); Pfam (Protein family); KOG/COG (Clusters of Orthologous Groups of proteins); Swiss-Prot (A manually annotated and reviewed protein sequence database); KO (KEGG Ortholog database); GO (Gene Ontology).

### Differential expression analysis

Gene expression levels were estimated by RSEM (Li and Dewey, 2011) for each sample. Differential expression analysis of two conditions/groups was performed using the DESeq R package (1.10.1). DESeq provide statistical routines for determining differential expression in digital gene expression data using a model based on the negative binomial distribution (Anders and Huber, 2010). Genes with an adjusted *P* < 0.05 found by DESeq were assigned as differentially expressed. Gene Ontology (GO) enrichment analysis of the differentially expressed genes (DEGs) was implemented by the GOseq R packages based Wallenius non-central hyper-geometric distribution (Young et al., 2010), which can adjust for gene length bias in DEGs. KEGG (Kanehisa et al., 2008) is a database resource for understanding high-level functions and utilities of the biological system, such as the cell, the organism and the ecosystem, from molecular-level information, especially large-scale molecular datasets generated by genome sequencing and other high-throughput experimental technologies (http://www.genome.jp/kegg/). We used KOBAS (Mao et al., 2005) software to test the statistical enrichment of differential expression genes in KEGG pathways.

### Validation of differentially expressed genes by quantitative real-time PCR (qRT-PCR)

Total RNA of seed or pericarp tissues from 60 DAP was extracted as described above and genomic DNA contamination was digested using DNase I (Qiagen). First-strand cDNA was reverse transcribed from 0.5 μg total RNA using the Reverse transcription System (Promega). The cDNA templates were then diluted 20-fold prior to use. qRT-PCR was performed with a StepOnePlus^™^ Real-Time PCR Systems (Applied Biosystems) using SYBR Premix ExTaq^™^ (TaKaRa) according to the manufacturer's protocol. All of the specific primers used for qRT-PCR were designed with PRIMER5 software (PREMIER Biosoft, USA) and are listed in Table S8. The expression of each gene was analyzed in three biological replicates, each with three technical repetitions. Relative expression levels were normalized by expression level of the internal control gene elongation factor 1-alpha (EF1A) and were calculated using the 2−ΔΔCt method.

## Results

### Oil accumulation in *I. polycarpa* fruits

**Figure 1:**
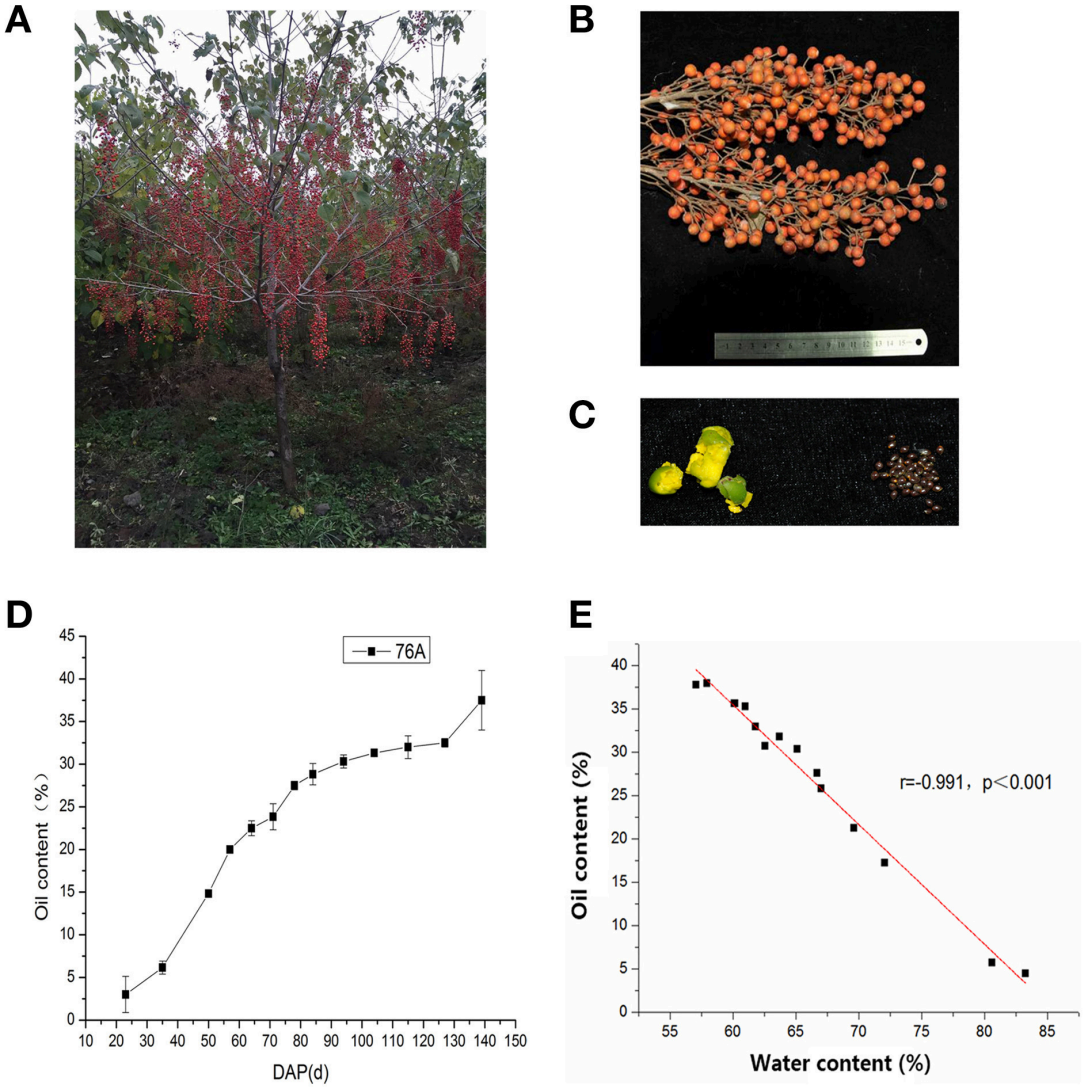
**Oil accumulation in ***I. polycarpa*** line 76A. (A)** View of the line 76A at harvest time. **(B)** Close view of the 76A fruits. **(C)** Separated seed and pericarp tissues from 76A fruits at 65 DAP (days post pollination). **(D)** Mean percentage of oil content (% dry weight) at different times during fruit development. Each point in the graphs represents the mean of three replicates ± SD. **(E)** Negative correlation of fruit oil content and water content.

In this experiment, we firstly collected the fruits of 73 *I. polycarpa* accessions and analyzed their oil content. We found that the fruits oil content was in the range of about 20–40% of dry weight (data not shown). Then, the accession 76A was selected for further research because of its proper height; relatively higher yield and fruit oil content (about 35%, Figures 1A,B,D). To assess the dynamic accumulation patterns of oils in developing fruits, we evaluated the 76A fruit oil contents at different developing stages (Figure 1D). Under field conditions, the fruits of 76A completed their development and maturation in approximately 160 DAP. Before 40 DAP, there was only slow accumulation of oil in the fruits (lower than 7% of dry weight). Between 40 DAP and 80 DAP, a significant increase in fruit oil content occurred (30% of dry weight), representing the most intense oil accumulation period. During the later developmental stages after 80 DAP, the oil content increased at a much lower rate but the water content still decreased. Interestingly, the oil content still increased at harvest time. Furthermore, it was found that there exist a strongly negative correlation between fruit oil content and water content (Figures 1D,E), suggesting a close relation between fruit dehydration and fruit lipid synthesis.

**Figure 2:**
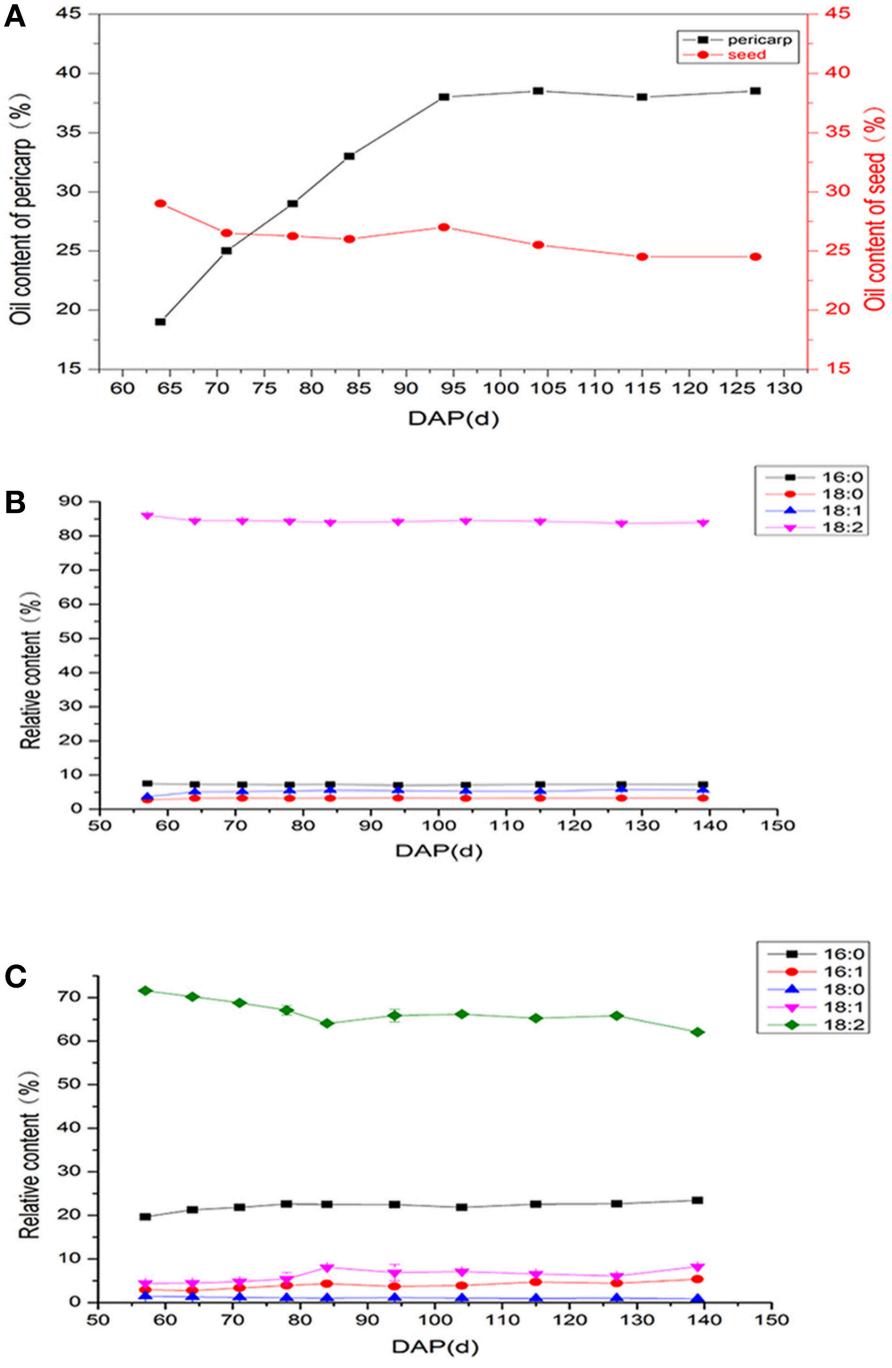
**Changes in the oil content and fatty acid composition of seed and pericarp during fruit development. (A)** Patterns of oil accumulation in the developing seed and pericarp. **(B)** Changes in fatty acid composition of seed during fruit development. **(C)** Changes in fatty acid composition of pericarp during fruit development. Values are means (±SD) of three biological replicates. C16:0, palmitic acid; C16:1 palmitoleic acid; C18:0, stearic acid; C18:1, oleic acid; C18:2, linoleic acid.

To further dissect their relative contributions to total fruit lipid accumulation, we separated seed and pericarp tissues from fruits at different developmental stages and examined their oil content respectively (Figures 1C, 2A). It should be noted that fruits before 65 DAP were not collected. Because at these stages, the fruits were tiny and have high water content (>80% of fresh weight) and seed and pericarp tissues can hardly be separated precisely. As shown in Figure 2A, the seeds had already reached a high oil content at the early stage of fruit development (before 65 DAP), when pericarp oil had not yet accumulated. It suggests that the seeds made the major contribution to oil biogenesis from early fruit growth stages. After 65 DAP, the pericap occupied the continuing increase in fruit oil content during the following ripening process, while the relative oil content in seeds showed slight decreases. However, the water content of fruits decreased and dry matter still increased in the seeds after 65 DAP, we believe that active lipid synthesis still occurred in the seeds. At harvest time, the pericarp showed much higher oil content than the seed (Figure 2A), implying their different ability for lipid accumulation.

Previous studies have shown that *I. polycarpa* fruits consisted of 5 fatty acids including palmitic acid (C16:0), palmitoleic acid (C16:1) stearic acid (C18:0), oleic acid (C18:1), and linoleic acid (C18:2), with >70% being C18:2. In this research, we further analyzed their relative profiles in seed and pericarp tissues (Table 1). Unexpectedly, the fatty acid composition showed great differences between the two tissues. The major fatty acid in both seed and pericarp was C18:2, which accounted for 83.92 and 62.08% of the total fatty acids, respectively. In addition, the relative abundances of C16:0 and C16:1 was higher in pericarps than in seeds, indicating that different fatty acid metabolism may exist in the two tissues. To ascertain whether the developmental process influenced the fatty acid profiles of the two tissues, we examined the percentage of individual fatty acids at various stages (Figures 2B,C). Interestingly, except for the C18:2 content in pericarps showing gradual and marginal decreases, the relative ratio of other fatty acids did not exhibit any important changes during the detected developmental process, both in seeds and pericarps. These unchanging trends of fatty acid profiles are very different with the storage component accumulation in other wood plants, such as oil palm, olive, and tung tree. In these plants, their major fatty acid composition increased significantly accompanied with fruit development processes (Dussert et al., 2013; Munoz-Merida et al., 2013; Galli et al., 2014).

**Table 1:** **Fatty acid compositions of seed and pericarp at harvest time**.

**Fatty acid%**	**16:0%**	**16:1%**	**18:0%**	**18:1%**	**18:2%**
Seed	7.22 ± 0.04	0	3.23 ± 0.02	5.62 ± 0.09	83.92 ± 0.07
Pericarp	23.42 ± 0.04	5.36 ± 0.05	0.88 ± 0.01	8.26 ± 0.08	62.08 ± 0.05

*Values are means (±SD) of three biological replicates*.

### RNA sequencing and gene annotation of *I. polycarpa* fruits expressed genes

In view of the great difference of oil content and fatty acid composition between seed and pericarp in *I. polycarpa* fruits, we anticipate that it provides an excellent platform to elucidate how the synthesis of lipid storages is regulated and directed into specific tissues. Such information is valuable for work aimed at either increasing the oil content in present oil crops or searching for novel ways for oil to accumulate in tissues that normally store little oil.

In recent years, advances in low cost next-generation sequencing technology have made RNA sequencing become an effective choice for fatty acid and TAG biosynthesis regulation research (Marchive et al., 2014). Based on the above results, seed and pericarp tissues from the fruits with high rates of lipid accumulation at 60 DAP were chosen for comparative transcriptome analysis to better explore the molecular regulatory mechanism underlying oil synthesis.

A total of six cDNA libraries were constructed from seed and pericarp RNA samples, each with three biological replicates. Then the libraries were respectively sequenced by Illumina paired-end sequencing technology. The number of resulting raw reads, clean reads, clean bases, Q20 and GC content of each sample can be seen in Table 2. Totally, 356707894 clean reads corresponding to more than 53G bases were generated after removing low quality reads and adaptor sequences. The average Q20 was 97% and GC content was about 44%. All the raw data of RNA-seq have been submitted to the NCBI Sequence Read Archive (SRA; http://www.ncbi.nlm.nih.gov/sra/) under the accession number SRX1625819 for seed and SRX1625805 for pericarp, respectively.

**Table 2:** **Raw data and valid data statistics of RNA sequencing**.

**Sample**	**Raw reads**	**Clean reads**	**Clean bases**	**Error(%)**	**Q20(%)**	**Q30(%)**	**GC(%)**
Seed_1	49148972	47754540	7.16G	0.01	97.06	92.78	44.09
Seed_2	56967492	55161192	8.27G	0.01	97.22	93.1	44
Seed_3	66322844	64349612	9.65G	0.01	97.15	93.03	44.24
Pericarp_1	49186548	48287202	7.24G	0.02	96.81	92.25	44.03
Pericarp_2	74810700	73021172	10.95G	0.01	97.02	92.77	43.41
Pericarp_3	69871142	68134176	10.22G	0.01	97.08	92.87	43.4

Because there is currently no reference genome sequence for *I. polycarpa*, the Trinity assembler (Grabherr et al., 2011) was used for *de novo* assembly of the clean reads. A total of 164152 transcripts were assembled with N50 lengths of 1771 bp. Transcripts were further assembled into unigenes, yielding 120126 unigenes with a mean length of 652 bp and N50 length of 1057 bp, creating an initial reference transcriptome. The lengths of these unigenes varied from 201 to 16,722 bp. There were 80221 unigenes (66.28%) ranging in length from 201 to 500 bp, 20787 unigenes (17.17%) ranging in length from 501 to 1000 bp, 11375 unigenes (9.39%) ranging in length from 1000 to 2000 bp, and 7743 unigenes (6.39%) >2000 bp in length (Figure S1).

To identify the putative functions of genes, these assembled unigenes were compared against the non-redundant protein sequences available at various databases with an E-value threshold of 10^−5^, including NCBI non-redundant protein (Nr) database, Swiss-Prot protein database (SwissProt), Clusters of Orthologous Groups (COG) database, Gene Ontology (GO), and Kyoto Encyclopedia of Genes and Genomes (KEGG). Of 121026 unigenes, 57908 (48.2%) unigenes were annotated in at least one of the above public database (Table S1). All unigenes were aligned against the NR protein database of GenBank using BLASTX. The distribution of hits obtained against entries for other plants within the NCBI database was used to get a descriptive view of the newly generated dataset. The best hit from each annotated sequence was calculated and is presented in Figure S2. A majority of the best hits were from *Populus trichocarpa*, a wood plant (72.9%). The second most frequent species was *Medicago trunctula* (*4.1%*), followed by *Vistis vinifera* (*2.7%*) and *Jotropha curcas* (2.0%), as shown in Figure S2.

The Gene Ontology (GO) annotation for the assembled unigenes was used to categorize the functions of the predicted unigenes. In total, 28212 unigenes were assigned to 3 main GO categories and 46 classifications (Figure S3). It was shown that metabolic process, cellular process, binding, and catalytic activity are the most dominant categories involving more than 20,000 unigenes, but only a few of genes were associated with terms such as cell killing and extracellular matrix part. In the category biological process, 1276 unigenes were assigned as lipid metabolic process or lipid transport function, respectively.

To further assess the validity and integrity of the transcriptome libraries, unigenes annotated in the NR database were aligned to the KOG database to classify potential functions. In total, 12168 unigenes were aligned to the 26 KOG classifications (Figure S4). The majority of unigenes were assigned to the general functional prediction, followed by post-translational modification, protein turnover, chaperone, translation, or signal transduction. Interestingly, there was about 4.8% unigenes assigned as lipid transport and metabolism function.

**Figure 3:**
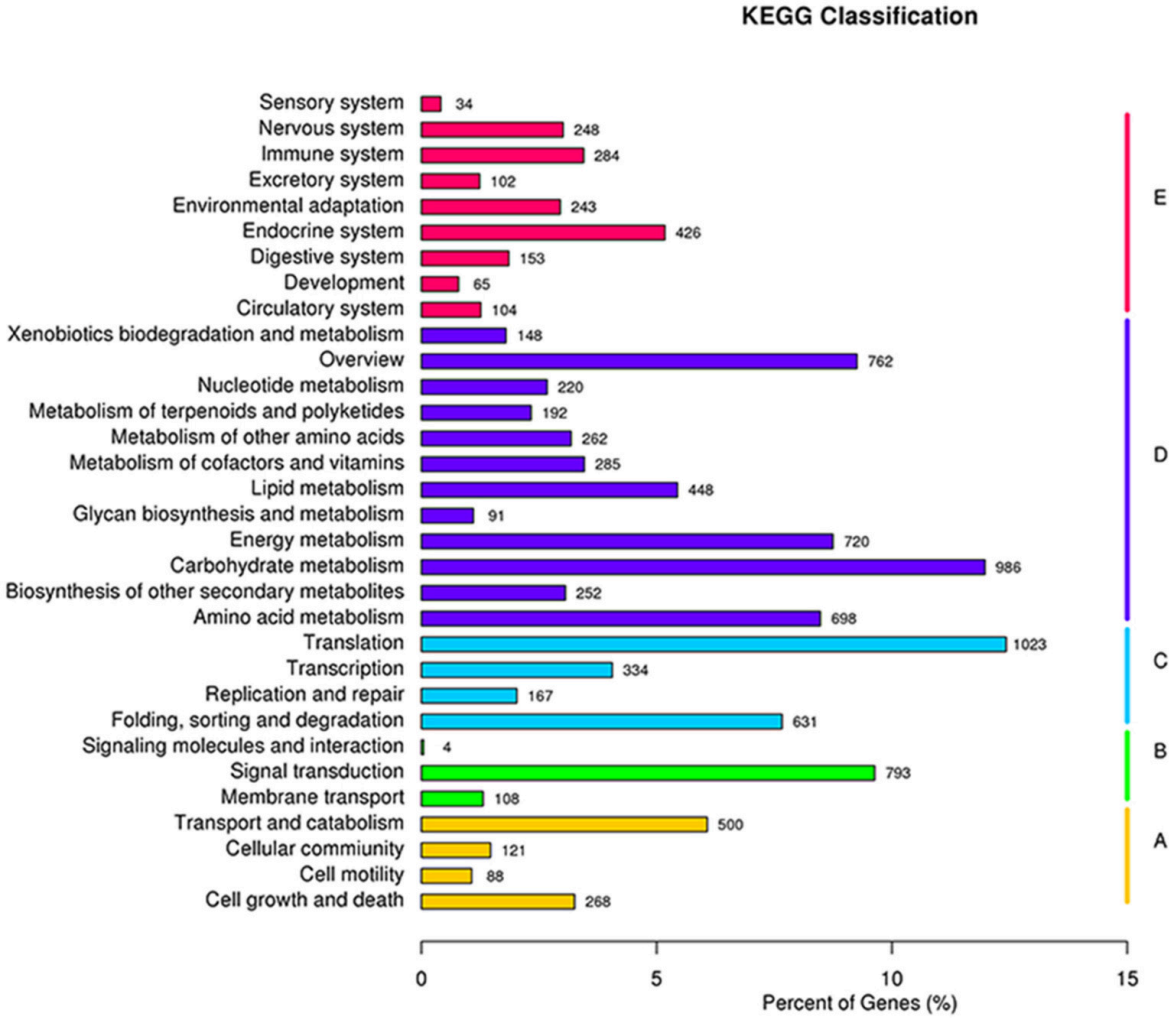
**Distribution of unigenes in different KEGG pathways**.

To understand the interaction of genes and metabolic biological functions, 8232 unigenes with significant BLASTX matches (E-value threshold of 10^−5^) in the KEGG database were assigned to 268 pathways (Table S2). As observed in Table S2 and Figure 3, the unigenes are widely distributed in distinct metabolic pathways, confirming the large coverage of the transcriptome obtained. A total of 448 and 986 unigenes were assigned to the lipid metabolism and carbohydrate metabolism respectively, which will provide a valuable tool for the study of lipid biosynthesis. In lipid metabolism genes, contigs that exhibited high similarity to genes encompassed all steps of the lipid synthesis pathway. Of which, 50 are related to fatty acid biosynthesis, 77 unigenes are classified as glycerolipid metabolism, 61 are related to the biosynthesis of unsaturated fatty acids, 27 are from fatty acid elongation pathway, 90 are from fatty acid degradation pathway, 32 are belonged to linoleic acid metabolism and 15 are involved in cutin, suberine, and wax biosynthesis (Table S2).

### Differential gene expression analysis in seed and pericarp

To fully explore the genes expression between seed and pericarp, the clean reads from each library were mapped to the reference transcriptome for profiling the expression of unigenes. Finally, we were able to estimate the abundance of each unigene in the seed and pericarp tissues. Many of the top 50 most highly expressed genes in seeds were those coding for seed storage proteins belonging to the 2S albumin and late embryogenesis abundant proteins (Table S3). Interestingly, two lipid metabolism genes, fatty acid desaturase *FAD2* (the 11^th^), and a Patatin-like phospholipase (the 10^th^) were highly expressed in the seeds. An oleosin gene, which functions in the lipid assembly, was ranked as the 7^th^ highly expressed gene in seeds. Importantly, this gene was not expressed in the preicarp, indicating a seed specific lipid assembling mechanism. Other genes highly expressed in seeds also contain several heat shock proteins or dehydration responsive genes, suggesting their possible protection roles during seed maturation. When we further examined the top 50 most highly expressed genes in percarps, it is of great interest to find that 40% (20/50) of the genes were the same as in seeds, reflecting the similarity of the two tissues (Table S4). The most exciting finding came from the *FAD2* gene (the 6^th^ highly expressed gene in pericarps). As *FAD2* is well-known for its role in fatty acid desaturation process, *FAD2* highly expressed in both the seed and pericarp may suggest that this gene is responsible for the high linoleic aicd content in the *I. polycarpa* fruit. Similarly, heat shock proteins or dehydration responsive genes were also shown to be highly represented in the pericarp, consistent with the above result that fruit oil content is negatively correlated with fruit water content (Figure 1). The pericarp also possesses highly expressed transcripts encoding protein homolog that was suggested to be present on the large lipid droplets in vegetable cells, termed as LIPID DROPLET-ASSOCIATED PROTEINs (Horn et al., 2013), implying a different lipid assembly mechanism in pericarp compared with seeds.

**Figure 4:**
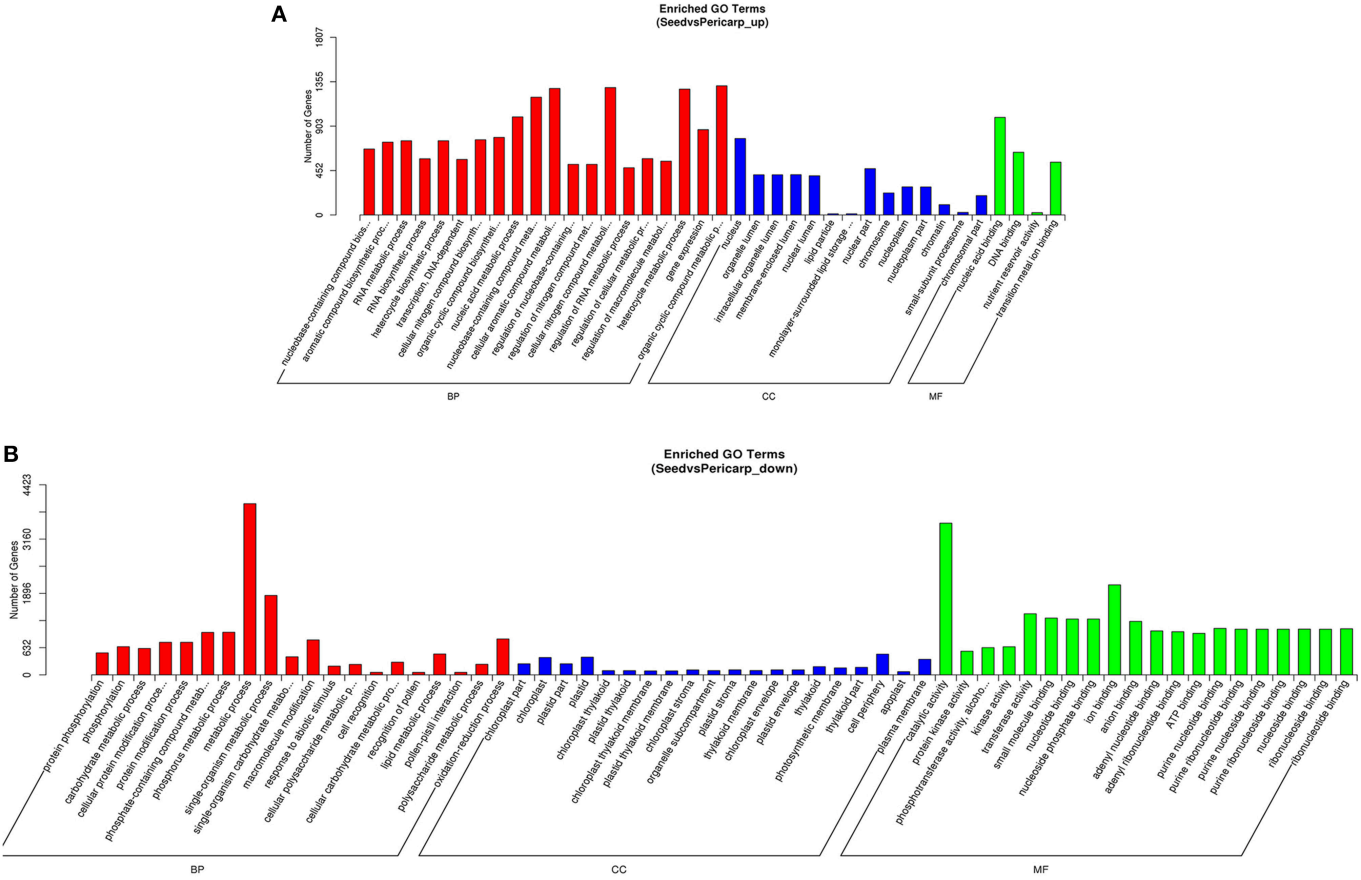
**GO enrichment analysis of differentially expressed genes between seed and pericarp. (A)** GO enrichment of seed up regulated genes. **(B)** GO enrichment of seed down regulated genes.

Also, the FPKM (expected number of Fragments Per Kilobase of transcript sequence per Millions base pairs sequenced) values were statistically calculated to select differentially expressed unigenes by using the DESeq method (Anders and Huber, 2010). The resulting P values were adjusted using the Benjamini and Hochberg's approach for controlling the false discovery rate. Genes with an adjusted *P* < 0.05 found by DESeq were assigned as differentially expressed. In total, we found 19121 differentially expressed genes, with 8589 up and 10532 down expressed in seed compared with pericarp (Table S5). To explore the genes' functions, GOseq and KOBAS (Mao et al., 2005; Young et al., 2010) software were used to test the statistical enrichment of differential expression of genes in Gene Ontology and KEGG pathways respectively. In GO analysis, the term nucleic acid binding (8.00E-12), lipid particle or monolayer-surrounded lipid storage body (*p* = 4.90E-06), cellular nitrogen compound biosynthetic process (7.59E-09), or RNA metabolic process (4.14E-09) showed over-representation in seed up regulated genes (Figure 4A). While in seed down regulated genes, genes involved in chloroplast (1.28E-31), catalytic activity (1.16E-30), and protein kinase activity (1.58E-30) were mostly overrepresented (Figure 4B). In KOBAS analysis, pathways of spliceosome (*q* = 0.000891338), plant hormone signal transduction (*q* = 0.019519796), regulation of autophagy (*q* = 0.040526256) showed statistical enrichment in seed up regulated genes (Figure S5). While in seed down regulated genes, the first enrichment pathway is plant hormone signal transduction (*q* = 0.337033857), however, no pathways showed statistical enrichment (Figure 4B). Together, these results showed no significant difference at lipid or fatty acid pathways level between seed and pericap, except for lipid particle genes in seeds. However, if we have a close look at the KEGG annotated differentially expressed genes, we could find that many genes involved in fatty acid elongation, biosynthesis of unsaturated fatty acids, fatty acid metabolism, fatty acid degradation, fatty acid biosynthesis and glycerolipid metabolism showed differential expression (Table 3). These genes should be related to the oil content and fatty acid composition difference between the seed and pericarp tissues. Their specific functions need more detailed investigations.

**Table 3:** **KEGG analysis of differentially expressed genes in lipid metabolism**.

**#Term**	**Database ID**	**Input number**	**Background number**	***P*-Value**
Fatty acid elongation	ko00062	13	27	0.1782944
Biosynthesis of unsaturated fatty acids	ko01040	25	61	0.2233141
Fatty acid metabolism	ko01212	42	112	0.2825529
Fatty acid degradation	ko00071	30	96	0.6531182
Fatty acid biosynthesis	ko00061	20	50	0.2837703
Sphingolipid metabolism	ko00600	15	28	0.095517
Glycerolipid metabolism	ko00561	31	77	0.214536
Glycerophospholipid metabolism	ko00564	25	70	0.4204502

### Detailed analysis of differentially expressed genes involved in lipid and fatty acid synthesis

Among higher plants, the *Arabidopsis* proteome is the most completely annotated and experimentally verified, and includes a recent update of information on over 600 genes involved in lipid metabolism (Li-Beisson et al., 2013). To assign functions to the *I. polycarpa* unigenes, we annotated the genes against the *Arabidopsis* lipid metabolism related genes (TAIR 10.0) with BLASTX. Altogether, 549 unigenes correlated to over 400 *Arabidopsis* lipid genes were expressed in *I. polycarpa* fruits (Table S6). Among them, 198 showed differential expression between seed and pericarp, with 136 up in pericarp and 52 up in seed. Furthermore, the genes were manually annotated and categorized using *Arabidopsis* information on the basis of their biochemical pathway and subcellular localization (Table S7).

#### FA biosynthesis related genes

Plant *de novo* fatty acid (FA) biosynthesis occurs in plastids and is performed by a fatty acid synthase dissociable complex of monofunctional enzymes. Briefly, the pyruvate dehydrogenase (PDH) complex generates acetyl-CoA, the building block used for fatty acid production. The first step of FA biosynthesis is the conversion of acetyl-CoA to malonyl-CoA by acetyl-CoA carboxylase (ACC). The malonyl group is then transferred from CoA to the acyl carrier protein (ACP), and the condensation between malonyl-ACP and acetyl-CoA, catalyzed by the fatty acid synthase complex, is the first of a series of sequential reactions of condensation, reduction, and dehydration adding two-carbon-units to the elongating acyl chain. Acyl chains are ultimately hydrolyzed by acyl-ACP thioesterases that release fatty acids (Bates et al., 2013; Li-Beisson et al., 2013). We found 24 differentially expressed genes involved in almost all part of fatty acid biosynthesis, including 2 *PDH* E1 subunit, 4 *ACC* subunit, *ACP5, KAS1* (3-ketoacyl-acyl carrier protein synthase I), *ENR* (Enoyl-ACP Reductase 1), and an [acyl-carrier-protein] S-malonyltransferases (Table 4). Other genes, such as *ATP citrate lyase* and several *KCS* (3-ketoacyl-CoA synthase) may be required for fatty acid synthesis or elongation outside the plastid. Importantly, all the genes were exclusively highly expressed in pericarp, suggesting the possibility that they were co-regulated and responsible for the higher oil content in pericarp.

**Table 4:** **Differentially expressed fatty acid synthesis-related genes**.

**Unigene_id**	**Seed_readcount**	**Pericarp_readcount**	**log_2_ Fold change**	***P*-value**	**Arabidopsis ID**	**Gene symbols**	**Annotation**
c62153_g1	141.9154829	334.5213088	−1.2371	1.18E-14	AT1G60810	ACLA-2	ATP-citrate lyase A-2
c68970_g1	1542.608536	3748.684993	−1.281	9.26E-29	AT1G09430	ACLA-3	ATP-citrate lyase A-3
c70118_g1	1959.901206	5445.798989	−1.4744	7.90E-41	AT5G49460	ACLB-2	ATP citrate lyase subunit B 2
c32684_g1	1523.776186	4934.041629	−1.6951	3.38E-13	AT5G27200	ACP5	Acyl carrier protein 5
c67420_g2	796.2114592	2268.835501	−1.5107	1.96E-37	AT5G15530	BCCP2	Biotin carboxyl carrier protein 2
c61853_g2	88.1858401	596.7740173	−2.7586	1.01E-38	AT5G16390	BCCP1	Chloroplastic acetylcoenzyme A carboxylase 1
c69864_g1	2028.977293	8731.196431	−2.1054	1.39E-80	AT2G38040	CAC3	Acetyl-CoA carboxylase carboxyltransferase alpha subunit
c70948_g1	367.0003529	1303.216277	−1.8282	5.55E-48	AT2G38040	CAC3	Acetyl-CoA carboxylase carboxyltransferase alpha subunit
c56856_g1	449.9052925	2060.035437	−2.195	1.14E-19	AT1G34430	EMB3003	2-oxoacid dehydrogenases acyltransferase
c63620_g1	865.8685168	2032.82467	−1.2313	1.11E-25	AT2G30200	EMB3147	[acyl-carrier-protein] S-malonyltransferases
c57663_g1	1628.971486	3528.016082	−1.1149	1.90E-23	AT5G46290	KASI	3-ketoacyl-acyl carrier protein synthase I
c34901_g1	0.341365051	52.15706738	−7.2554	1.41E-22	AT1G24470	KCR2	Beta-ketoacyl reductase 2
c65321_g2	26.53949898	338.0346742	−3.671	1.40E-33	AT1G01120	KCS1	3-ketoacyl-CoA synthase 1
c46394_g1	0.313028125	251.4243765	−9.6496	4.81E-58	AT1G04220	KCS2	3-ketoacyl-CoA synthase 2
c46394_g2	14.62105482	100.8772148	−2.7865	4.14E-20	AT5G43760	KCS20	3-ketoacyl-CoA synthase 20
c65184_g1	546.3901799	1629.254612	−1.5762	3.79E-39	AT5G43760	KCS20	3-ketoacyl-CoA synthase 20
c65711_g2	43.63627876	16779.04481	−8.5869	0	AT1G07720	KCS3	3-ketoacyl-CoA synthase 3
c58655_g1	2308.650235	1007.470925	1.1963	1.78E-26	AT2G16280	KCS9	3-ketoacyl-CoA synthase 9
c66011_g1	909.2469392	5204.84245	−2.5171	1.37E-37	AT3G25860	LTA2	2-oxoacid dehydrogenases acyltransferase
c63835_g2	195.394919	2105.371561	−3.4296	3.30E-90	AT2G05990	ENR1	ENOYL-ACP REDUCTASE 1
c63835_g1	467.1092453	1380.560582	−1.5634	5.65E-36	AT2G05990	ENR1	ENOYL-ACP REDUCTASE 1
c6342_g1	3249.471207	12297.61105	−1.9201	1.08E-68	AT1G01090	PDH-E1	Pyruvate dehydrogenase E1 alpha
c66590_g1	3491.019004	8386.041767	−1.2643	8.97E-13	AT1G30120	PDH-E1	Pyruvate dehydrogenase E1 beta
c67036_g2	1421.298497	11085.86725	−2.9634	5.27E-130	AT4G01900	PII	GLNB1 homolog

Previous researches have demonstrated that most of the genes involved in the core FA biosynthetic machinery share the same temporal transcription pattern and are co-regulated by the AP2 family transcription factor *WRI1* (Ruuska et al., 2002; Cernac and Benning, 2004; Baud et al., 2009; To et al., 2012; Marchive et al., 2014). We found two genes encode *WRI1* in the *I. polycarpa* fruit transcriptome (Table S5), of which, c68533_g2 showed over 10-fold higher expression in the pericarp than in the seed, implying that it is the potential target that improves the fatty acid synthesis related gene expression and increases oil content in the pericarp. In addition, we also found another 6 transcriptional factors corresponding to 7 genes which showed differential expression between seed and pericarp, including *LEC1* (*LEAFY COTYLEDON 1*), *LEC2* (*LEAFY COTYLEDON 2*), *ABI4* (*ABSCISIC ACID INSENSITIVE 4*), *ABI3* (*ABSCISIC ACID INSENSITIVE 3*), *FUS3* (*FUSCA 3*), and *MYB30*. In previous studies, the transcription factors LEC1, LEC2, ABI4, ABI3, and FUS3 have been shown to participate in seed maturation and seed oil synthesis by up-regulating the expression of *WRI1* in *Arabidopsis* seeds (Baud et al., 2007; Gutierrez et al., 2007; Mu et al., 2008; Baud and Lepiniec, 2010). However, in this study, these 5 genes were specifically expressed in the seed but not expressed in the pericarp (Table 5), and *WRI1* expression in seed was lower than in pericarp, indicating that the higher expression of *WRI1* in the pericarp was independent of the conserved *LEC1, LEC2, ABI4, ABI3*, and *FUS3* pathway.

**Table 5:** **Differentially expressed transcription factor genes**.

**Unigene_id**	**Seed_readcount**	**Pericarp_readcount**	**log_2_ Fold change**	**P-value**	**Arabidopsis ID**	**Gene symbols**	**Annotation**
c68829_g1	2164.5335	6.100976694	8.4708	0	AT3G24650	ABI3	AP2/B3-like transcriptional factor family protein
c61821_g1	397.5042262	6.922591832	5.8435	4.99E-119	AT2G40220	ABI4	Integrase-type DNA-binding superfamily protein
c58701_g1	1891.2354	754.3840568	1.326	2.99E-31	AT3G28910	ATMYB30	myb domain protein
c65081_g1	709.9822534	0.420171347	10.723	7.88E-216	AT3G26790	FUS3	AP2/B3-like transcriptional factor family protein
c42649_g1	4.750855347	0	Inf	0.0055798	AT3G26790	FUS3	AP2/B3-like transcriptional factor family protein
c63503_g1	26.057581	0	Inf	1.34E-13	AT1G21970	LEC1	Histone superfamily protein
c67058_g1	6416.3696	15.09446955	8.7316	0	AT1G28300	LEC2	AP2/B3-like transcriptional factor family protein
c68533_g2	244.8903808	2724.703788	−3.4759	3.09E-58	AT3G54320	WRI1	Integrase-type DNA-binding superfamily protein

#### TAG assembly related genes

Two metabolic pathways for the production of TAGs have been elucidated: an acyl-CoA-dependent pathway and an acyl-CoA-independent pathway, both occurring in the ER. In the acyl-CoA dependent pathway, commonly known as the Kennedy pathway, acyl-CoA is used as a substrate for the serial incorporation of three acyl groups into the glycerol backbone. This pathway is dependent on enzymes such as glycerol-3-phosphate acyltransferase (GPAT), lysophosphatidic acid acyl transferase (LPAT), and phosphatidic acid phosphatase (PAP), resulting in the formation of diacylglycerol (DAG). Finally, diacylglycerol acyltransferase (DGAT) transfers an acyl group from acyl-CoA to *sn* -3 of DAG to form TAG (Bates et al., 2013; Li-Beisson et al., 2013). Totally, we found 9 unigenes coding for GPAT, 6 for LPAT, 3 for DGAT and 6 for PAP expressed in *I. polycarpa* fruit. Among them, 11 genes showed differential expression between seed and pericarp (Table 6). In seeds, *LPAT2* and *GPAT7* showed higher expression, while in pericarps, *GPAT2, GPAT6*, and *LPAT5* showed higher expression. The results suggest that seed and pericarp may use different genes for TAG synthesis. Furthermore, *glycerol-3-phosphate kinase, 6-phosphogluconate dehydrogenase* and *glycerol-3-phosphate dehydrogenase*, genes required for glycerol-3-phosphate metabolism (a TAG precursor), and *DGAT2*, were all increased in the pericarp (Table 6). Increasing the expression of such genes may lead to a greater flux of intermediates through the Kennedy pathway and alter TAG accumulation (Jako et al., 2001; Nandi et al., 2004). The acyl-CoA-independent pathway involves phospholipid/diacylglycerol acyltransferase (*PDAT*) to produce TAG. We did find a homolog of *Arabidopsis PDAT1* expressed in *I. polycarpa* fruit but it expression level was similar in seed and pericarp. These results suggest that the acyl-CoA-independent pathway may not be the most important pathway for the different synthesis of TAGs between seed and pericarp. After biosynthesis, pools of TAGs can be stored as a form of oil bodies surrounded by a single phospholipid monolayer and abundant amphipathic proteins such as oleosin, caleosin, and steroleosin in mature seeds (Chapman et al., 2012). A caleosin gene c65613_g2, showed seed preferred expression, suggesting its role in seed oil body formation. Surprisingly, an oleosin gene encoded by c61328_g1, showed even higher expression in pericarp than in seed. Its role in oil body formation needs to be further investigated.

**Table 6:** **Differentially expressed TAG synthesis-related genes**.

**Unigene_id**	**Seed_readcount**	**Pericarp_readcount**	**log_2_ Fold change**	***p*-value**	**Arabidopsis ID**	**Gene symbols**	**Annotation**
c70071_g1	869.226309	1997.196942	−1.2002	1.29E-25	AT3G07690		6-phosphogluconate dehydrogenase
c70227_g2	12.74288607	296.9678234	−4.5425	3.08E-51	AT5G55380		MBOAT (membrane bound O-acyl transferase)
c61328_g1	6685.327454	30741.27179	−2.2011	8.06E-48	AT5G07600		Oleosin family protein
c30787_g1	289.8070546	721.2940035	−1.3155	1.86E-22	AT2G32260	ATCCT1	Phosphorylcholine cytidylyltransferase
c53647_g1	0	85.70741161	#NAME?	1.85E-35	AT1G02390	ATGPAT2	glycerol-3-phosphate acyltransferase 2
c60624_g1	2.559685658	30.6511831	−3.5819	1.49E-09	AT2G38110	ATGPAT6	glycerol-3-phosphate acyltransferase 6
c63031_g1	11.12228479	83.42098821	−2.907	2.05E-17	AT2G38110	ATGPAT6	glycerol-3-phosphate acyltransferase 6
c65209_g1	672.0532782	31.12270407	4.4325	2.58E-129	AT5G06090	ATGPAT7	glycerol-3-phosphate acyltransferase 7
c51519_g1	556.8863812	1168.71624	−1.0695	2.16E-18	AT1G15080	ATPAP2	Lipid phosphate phosphatase 2
c65613_g2	12110.20419	55.50632122	7.7694	0	AT4G26740	ATS1	Caleosin
c50582_g1	1124.836834	4951.865584	−2.1383	5.85E-39	AT3G51520	DGAT2	Diacylglycerol acyltransferase 2
c43324_g1	1085.918302	2207.943503	−1.0238	4.48E-13	AT5G40610	GPDHp	NAD-dependent glycerol-3-phosphate dehydrogenase
c50600_g2	42.81189121	8.263522421	2.3732	5.20E-09	AT3G57650	LPAT2	Lysophosphatidyl acyltransferase
c50600_g1	18.4340118	3.197232374	2.5275	0.0001325	AT3G57650	LPAT2	Lysophosphatidyl acyltransferase
c62123_g1	165.5833182	350.6045949	−1.0823	1.59E-11	AT3G18850	LPAT5	Lysophosphatidyl acyltransferase 5
c65924_g1	1023.941515	2936.312625	−1.5199	3.85E-40	AT1G12640	LPCAT1	LYSOPHOSPHATIDYLCHOLINE ACYLTRANSFERASE 1
c64318_g1	38.2027205	263.7547832	−2.7874	6.41E-41	AT3G02600	LPP3	Lipid phosphate phosphatase 3
c63860_g1	177.6129011	422.4644063	−1.2501	2.04E-16	AT3G02600	LPP3	Lipid phosphate phosphatase 3
c59976_g1	447.1358946	3454.619686	−2.9497	1.33E-130	AT1G80460	NHO1	Glycerol kinase

#### Fatty acid modification related genes

The aforementioned results suggested that the seed and pericarp tissues showed significant differences in fatty acid compositions, especially in C16:0, C16:1, and C18:2 contents (Table 1). In *Arabidopsis*, it has been found that fatty acid compositions were systemically regulated by multiple fatty acid desaturation and lipid trafficking pathways. Fatty acid desaturation is regulated by genes encoding for the plastid localized fatty acid desaturase (*SSI2, FAD4, FAD5, FAD6, FAD7, FAD8*) and the ER localized fatty acid desaturases (*FAD2* and *FAD3*). As well, Acyl-ACP thioesterase (*FATA* and *FATB*) and long-chain acyl-CoA synthetase (*LACS*) are responsible for fatty acids release from plastid and their trafficking between plastid and ER (Bates et al., 2013; Li-Beisson et al., 2013). Here, we totally annotated 19 fatty acid modifying related genes that showed differential expression between seed and pericarp (Table 7). Since the *I. polycarpae* specially had very high C18:2 in its fruits, we firstly focus on the *FAD2* gene, which has been well-known for its role in C18:2 syntheses (Hernandez et al., 2016). Among the assigned 549 fatty acid genes, we found four genes encoding homologs of the *Arabidopsis FAD2* gene. Among them, three genes showed differentially expression between seed and pericarp. Interestingly, c56614_g1 has been found in the top 50 highly expressed unigenes, both in seed and pericarp, indicating a highly basic C18:2 synthesis ability. However, two other *FAD2* homologs c63420_g2 and c50543_g1, exhibited opposite expression patterns. c63420_g2 was 13.5 times higher in pericarp than in seed, while c50543_g1 showed 74.8 times higher in seed than in pericarp. It suggested that pericarp and seed used both identical and distinct *FAD2* genes for C18:2 syntheses, possibly in a tissue specific manner. Similarly, we found two homologs of *SSI2* gene, which is responsible for C18:1 synthesis. c55775_g1 showed about 8 times higher in pericarp, while c65723_g1 displayed 5.3 times higher in seed. Consistent with the specifically higher C16:1 content in pericarp, two homologs of *FAD5*, which had been reported to be responsible for C16:1 synthesis (Barkan et al., 2006), were expressed higher in the pericarp. We could not detect 16:3 or 18:3 fatty acids in the fruits. In consistent, *FAD3*, gene for C18:3 syntheses in ER, was not detected both in both seed and pericarp. Meanwhile, 2 and 4 unigenes encoding homologs of *FAD7* and *FAD8*, which is responsible for the synthesis of 16:3 and 18:3 fatty acids in plastids (Roman et al., 2015), also showed higher expression in the pericarp. However, because we could not detect C16:3 and C18:3 fatty acids in the pericarp, their physiological roles need to be examined. Furthermore, pericarps have very high C16:0 acid (23.42 ± 0.04) compared with seeds (7.22 ± 0.04). Two classes of thioesterases designated *FATA* and *FATB* has been reported to be responsible for the hydrolysis of unsaturated and saturated acyl-ACPs, respectively, and thus determine in large part the chain length and saturated FA content of plant oils such as C16:0 and C18:1(Belide et al., 2012; Moreno-Perez et al., 2012). We found ortholog genes of *Arabidopsis FATA* and *FATB*, but they showed similar expression in pericarp and seed, suggesting that another thioesterase may be responsible for C16:0 release. Indeed, two unigenes, c64914_g1 and c64322_g1 encoding thioesterase superfamily protein, showed higher expression in pericarp. Whether they have biochemical ability toward C16:0 needs further study.

**Table 7:** **Differentially expressed fatty acid composition modifying genes**.

**Unigene_id**	**Seed_readcount**	**Pericarp_readcount**	**log_2_ Fold change**	***p*-value**	**Arabidopsis ID**	**Gene symbols**	**Annotation**
c64914_g1	526.7088268	2559.98082	−2.2811	2.67E-25	AT2G22230		Thioesterase superfamily protein
c65723_g1	2587.191682	488.2888324	2.4056	3.03E-88	AT3G02630		Plant stearoyl-acyl-carrier-protein desaturase family protein
c64322_g1	1188.292891	11074.27656	−3.2203	1.87E-19	AT4G13050		Acyl-ACP thioesterase
c68244_g1	374.14807	60.63213358	2.6255	2.66E-48	AT4G04930	DES-1-LIKE	Fatty acid desaturase family protein
c63420_g2	134.579276	1816.370139	−3.7545	1.04E-157	AT3G12120	FAD2	Fatty acid desaturase 2 c56614_g1
c56614_g1	84822.70568	310962.0777	−1.8742	6.42E-21	AT3G12120	FAD2	Fatty acid desaturase 2
c50543_g1	1031.077652	13.8923392	6.2137	8.16E-68	AT3G12120	FAD2	Fatty acid desaturase 2
c67227_g1	245.8622471	1689.61515	−2.7808	1.22E-65	AT3G15850	FAD5	Fatty acid desaturase 5
c64519_g1	1.678515515	9.845952494	−2.5523	0.006779	AT3G15850	FAD5	Fatty acid desaturase 5
c59812_g1	2.41791947	68.56474717	−4.8256	1.12E-08	AT3G11170	FAD7	Fatty acid desaturase 7
c71381_g3	1246.245281	11517.87792	−3.2082	8.91E-171	AT3G11170	FAD7	Fatty acid desaturase 7
c56246_g1	1.36548739	26.31439815	−4.2684	7.68E-07	AT5G05580	FAD8	Fatty acid desaturase 8
c59828_g1	7.282176893	103.6470263	−3.8312	1.08E-15	AT5G05580	FAD8	Fatty acid desaturase 8
c59812_g2	3.670031969	16.59051141	−2.1765	0.001699	AT5G05580	FAD8	Fatty acid desaturase 8
c42915_g1	9.245356421	26.08789747	−1.4966	0.003294	AT5G05580	FAD8	Fatty acid desaturase 8
c70568_g1	1942.73209	4161.572697	−1.099	1.37E-23	AT4G23850	LACS4	LONG-CHAIN ACYL-COA SYNTHETASE 4
c24653_g1	14.9611795	138.4373451	−3.2099	6.18E-31	AT3G61580	SLD1	Fatty acid/sphingolipid desaturase
c55197_g1	303.6935583	785.5673169	−1.3711	2.32E-24	AT3G61580	SLD1	Fatty acid/sphingolipid desaturase
c55775_g1	4142.383194	31759.2726	−2.9386	7.79E-27	AT2G43710	SSI2	Stearoyl-acyl-carrier-protein desaturase

### Validation of differentially expressed genes by qRT-PCR

**Figure 5:**
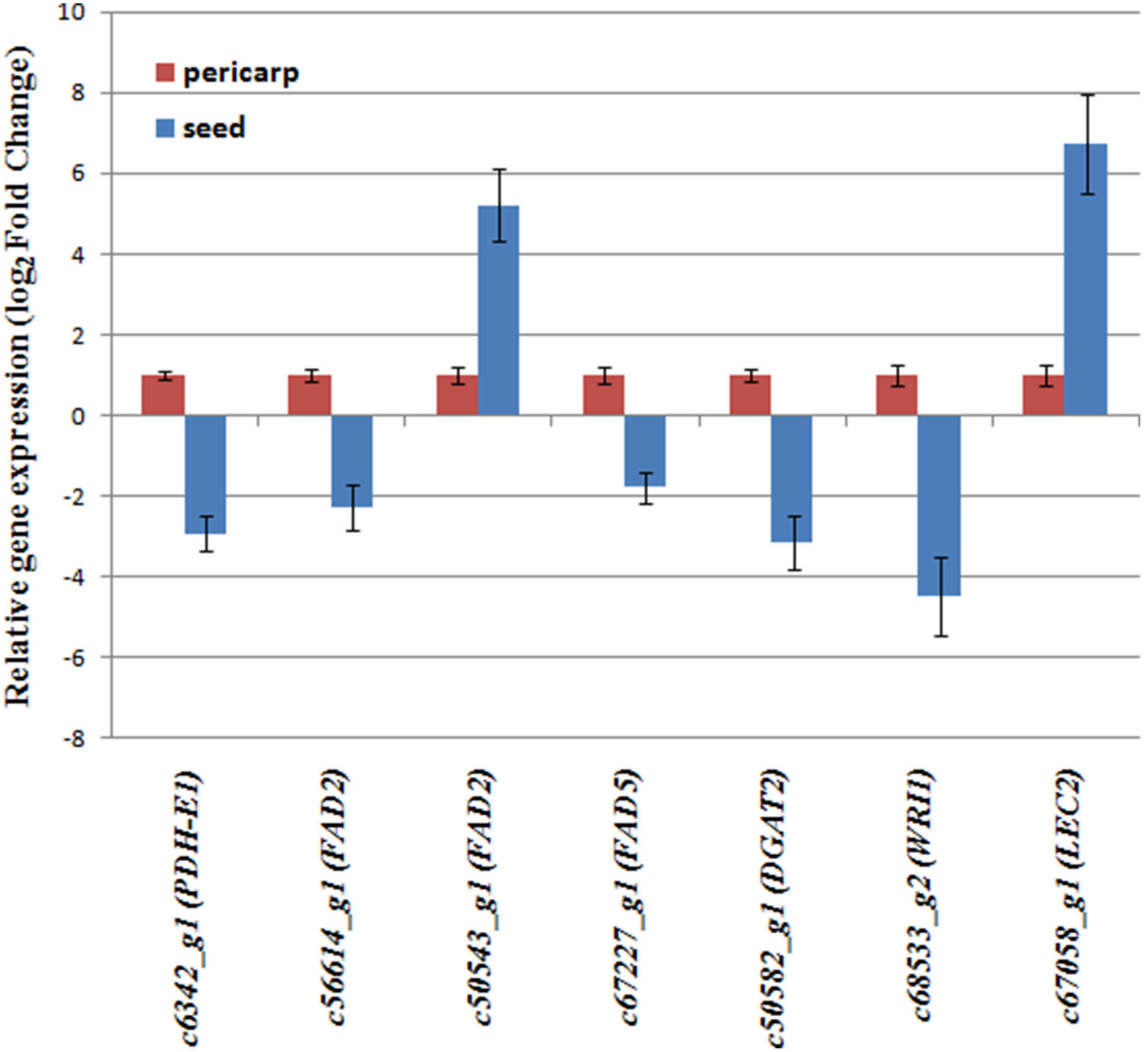
**qRT-PCR verification of selected differentially expressed genes**. Gene expression in the seed and pericarp at 60 DAP was examined with a StepOnePlus™ Real-Time PCR Systems (Applied Biosystems) using SYBR Premix ExTaq™ (TaKaRa) according to the manufacturer's protocol. Relative expression values were calculated using the 2−ΔΔCt method by using EF1A gene as an internal control and were given as mean of the normalized expression levels of three replicates. For comparison, the gene expression level in the pericarp was arbitrarily set as 1.

To confirm the reliability of the RNA-Sequencing results, the expression of seven candidate differentially expressed genes implicated in lipid metabolism were measured by qRT-PCR, including c6342_g1 (*PDH-E1*), c56614_g1 (*FAD2*), c50543_g1 (*FAD2*), c67227_g1 (*FAD5*), c50582_g1 (*DGAT2*), c68533_g2 (*WRI1*), and c67058_g1 (*LEC2*) (Figure 5). Our results showed that, at 60 DAP, although the fold changes in their expression between seed and pericarp detected by RNA sequencing and qRT-PCR did not match exactly, the expression patterns determined for all seven genes were consistent, confirming the reliability of the RNA-seq results (Figure 5). For example, for genes c56614_g1 (*FAD2*), c50543_g1 (*FAD2*), and c67227_g1 (*FAD5*), their expression showed −1.8742, 6.2137, and −2.7808 fold change (log_2_ ratio) between seed and pericarp in RNA sequencing results. In qRT-PCR, their expression changes were shown as −2.27, 5.25, and −1.86 fold respectively.

## Discussion

The *I. polycarpa*, a potential woody oil species for edible oil and biodiesel, has a high quality and quantity of oil and excellent adaptability to different growing conditions. In this work, kinetic patterns of oil contents and FA compositions were detected at different developing stages of *I. polycarpa* fruits, and the optimal for comparative deep transcriptomic analysis were determined. The transcriptomes of seed and pericarp were sequenced by using Illumina technology, and then the assembled unigenes were functionally annotated. Moreover, the differentially expressed genes for the enzymes and transcription factors involved in oil accumulation of developing fruits were screened by the DESeq method, and the role and regulation of some key genes was analyzed. Overall, the temporal accumulated patterns of oils and FA compositions, and the transcriptional profiles of transcriptional regulatory factors (*WRI1*) and metabolic enzymes associated with the biosynthesis of acetyl-CoA (*PDH, ACC, ACL*), FA (*KAS, ENR, ACP, FAD2, FAD5, FAD7, FAD8, SSI2*), TAG (*GPAT, LPAT, DGAT, PAP*), and oil body (caleosin, oleosin) were systematically analyzed in seed and pericarp, which will contribute to elucidate the molecular and metabolic mechanisms leading to different oil biosynthesis and fatty acid accumulation between the two tissues. We conclude from the results that *I. polycarpa* can be considered as a novel model that can significantly widen our understanding on how the synthesis of oil biosynthesis is regulated and directed in non-seed tissues. A better understanding of oil accumulation in fruits may present strategies for engineering oil accumulation in other vegetative tissues.

### Special characters of lipid accumulation in *I. polycarpa* fruits

*I. polycarpa* has high amount of C18:2 in both of the seed and the pericarp, whose contents showed great difference (83–63%). In addition, the pericarp contains a significant proportion of C16:0 and C16:1, whose content is low or absent in the seed (Table 1). More importantly, their oil accumulation trends and final oil content were also very different. Therefore, although developed in the same fruits, the two tissues have different biochemical or regulatory mechanism for lipid synthesis, which providing an excellent platform for comparative analysis. We suppose that the difference may lie in their different roles in plants. Oil stored in seeds is used to fuel post-germination growth of seedlings. However, the pericarp oil is used to attract animals and birds to transport and/or ingest the fruits, hence aiding seed dispersal, just like the surface wax of baberry (Simpson and Ohlrogge, 2016). Furthermore, we found that the relative ratio of fatty acids did not exhibit significant change during the detected developmental process, both in seed and pericarp. These trends of fatty acid profiles are very different with the storage component accumulation in other wood oil plants (Dussert et al., 2013; Munoz-Merida et al., 2013; Galli et al., 2014). For example, in oil palm, dramatic changes in FA composition have been reported during the development of both the endosperm and the mesocarp (Dussert et al., 2013). This suggests that *I. polycarpa* fruit also possess species specific mechanisms for lipid accumulation.

### Transcriptional regulation of fatty acid metabolism showed conserved and non-conserved patterns in *Idesia polycarpa* fruit

Current knowledge about oil synthesis is derived mainly from research on seeds (Baud and Lepiniec, 2010; Bates et al., 2013; Li-Beisson et al., 2013). In contrast, far less is known about the molecular basis of lipid metabolism in other non-seed tissues. Whether these important characteristics of seed oil synthesis are conserved and function in non-seed tissues remains to be elucidated (Chen et al., 2015; Divi et al., 2016; Xu and Shanklin, 2016).

During the last decade, extensive transcriptomic analyses have provided detailed expression patterns for genes involved in FA biosynthesis (Marchive et al., 2014). It has been shown that the rates of FA production were changing in a manner proportional to the transcript levels of genes encoding core FA biosynthetic enzymes in tissues analyzed, highlighting the importance of coordinated transcriptional regulation for the control of FA production (Baud et al., 2009; Bourgis et al., 2011; Tranbarger et al., 2011). In the *Arabidopsis* embryo, almost all genes involved in *de novo* FA synthesis show the same timing and pattern of expression. In contrast, the transcription of genes required for TAG assembly in the ER arises later and remains high during the maturation process (Ruuska et al., 2002). Using pyrosequencing, 7 million ESTs were generated from four stages of developing seeds of *Ricinus communis, Brassica napus, Euonymus alatus*, and *Tropaeolum majus*, which differ in their storage tissue for oil. Analysis of EST levels from these oilseeds revealed both conserved and distinct species-specific expression patterns for genes involved in the synthesis of glycerolipids and their precursors. Independent of the species and tissue type, ESTs for core fatty acid synthesis enzymes maintained a conserved stoichiometry and a strong correlation in temporal profiles throughout seed development (Troncoso-Ponce et al., 2011). The comparative analysis of the three TAG accumulating tissues of oil palm showed that transcriptional regulation plays a key role in the considerable differences in oil content and FA composition that exist between these tissues (Dussert et al., 2013). Oil palm can accumulate up to 90% oil in its mesocarp, the highest level observed in the plant kingdom. In contrast, the closely related date palm accumulates almost exclusively sugars. Interspecies transcriptome comparison analysis of the two palm revealed the high oil content in oil palm was associated with much higher transcript levels for all fatty acid synthesis enzymes. Unexpectedly, despite more than a 100-fold difference in flux to lipids, most enzymes of triacylglycerol assembly were expressed at similar levels in oil palm and date palm (Bourgis et al., 2011). Together, these data point to synthesis of fatty acids and supply of pyruvate in the plastid, rather than acyl assembly into triacylglycerol, as a major control over the storage of oil. In this study, our transcriptomic data found 24 pericarp highly expressed genes involved in almost all part of fatty acid biosynthesis, including 2 *PDH E1* subunit, 4 *ACC* subunit, *ACP5, KAS1, ENR*, [*acyl-carrier-protein*] *S-malonyltransferases* (Table 4), suggesting that such coordinated regulation of FA biosynthesis gene expression is conserved in plant oil-storing tissues, independent of their origin. In addition, this result, combined with previous publications, could lead to the hypothesis that the higher lipid synthesis or oil content in pericarp is mostly controlled by *de novo* FA synthesis.

The *WRINKLED1* transcription factor triggers the concomitant upregulation of genes involved in fatty acid production at the onset of the seed maturation phase (Cernac and Benning, 2004). This member of the APETALA2-ethylene-responsive element binding protein (AP2-EREBP) family controls the expression of at least 15 enzymes including pyruvate dehydrogenase, ACCase and members of the FA synthesis and glycolytic pathways (Baud et al., 2007). Thus, *WRI1* expression is pivotal in directing the carbon flux that enters the seed toward the synthesis of FAs. Oil content is reduced to the extent of 80% in *wri1* mutants (Cernac and Benning, 2004), while the seed-expressed *BnWRI1* or *ZmWRI1* orthologs have been confirmed to function in regulation of seed oil biosynthesis by their ability to complement *wri1* or to increase seed oil content (Liu et al., 2010; Pouvreau et al., 2011). In addition to controlling oil production in seeds, recent evidence indicates that *WRI1* is likely a major factor responsible for the extremely high oil content produced by oil palm mesocarp (Bourgis et al., 2011; Ma et al., 2013). Transcriptional profiling of oil palm mesocarp revealed >50-fold higher *WRI1* expression levels compared with date palm mesocarp, a closely related species that contains no oil. Consistent with data in developing seeds, genes encoding machinery for FA biosynthesis and pyruvate supply are up-regulated substantially in oil palm (an average of >13-fold). Moreover, transcriptome analysis of the developing oil palm mesocarp revealed that a *WRI1* gene was massively transcribed at the onset of oil accumulation and co-regulated with FA biosynthetic genes, suggesting *WRI1* may also regulate FA synthesis in non-seed tissues (Tranbarger et al., 2011). Consistently, we found that *WRI1* showed over 10-fold higher expression in the pericarp than in the seed, and that all its known targets were up-regulated, providing a strong indication that a *WRI1* ortholog plays a major role in oil accumulation in the pericarp. This also implies a remarkable similarity between regulation of fatty acid synthesis destined to oil in seeds and in non-seed tissues.

In *Arabidopsis, WRI1* is under the control of seed maturation master regulators such as *LEAFY COTYLEDON1* and *-2, FUSCA3* and *ABSCISIC ACID INSENSITIVE3* and *4* (Baud et al., 2007; Gutierrez et al., 2007; Mu et al., 2008; Baud and Lepiniec, 2010). However, no obvious orthologs to these genes were identified in oil palm mesocarp (Dussert et al., 2013; Ma et al., 2013), suggesting that *EgWRI1-like* is likely to control oil synthesis independently of the upstream factors that participate in seed development and may involve a possible fruit-specific regulatory cascade. Additionally, no sequences with significant similarity to *LEC2* and *FUS3* were found even in the embryo and endosperm transcriptomes of oil palm (Dussert et al., 2013). In contrast, we did found *LEC1, LEC2, ABI4, ABI3*, and *FUS3* homolog genes expressed in *I. polycarpa* seed, like in *Arabidopsis*. However, they showed absent or very low expression in pericarp, as in oil palm (Table 5). These results suggest that species and tissues specific network may have evolved to precisely control the expression of *WRI1* for fatty acid synthesis. The identification of the regulatory cascade that controls *WRI1* expression in fruit is our critical issue in the future research.

### Diversification of fatty acid composition modifying genes

Fatty acid desaturation contains two steps in plastid and ER, respectively. The first step is the formation of monounsaturated fatty acids from saturated fatty acids in plastids. The synthesis of fatty acids may be accomplished by producing 16:0-ACP fatty acids, which are hydrolyzed by acyl-ACP thioesterases (FATA and FATB) that release fatty acids from the ACP molecule to be transported to ER. However, the 18:0-ACP generated by FAS may be desaturated by stearoyl-ACP desaturase *SSI2* to produce unsaturated fatty acids C18:1 before being released from ACP and transported to the ER. The second step is the formation of unsaturated bonds on the monounsaturated fatty acids at specifically defined positions, which is catalyzed by enzymes located on the membranes of the endoplasmic reticulum and chloroplast, including *FAD2* and *FAD6*, which desaturates oleic acid (18:1) to form linoleic acid (18:2), and *FAD3, FAD7*, and *FAD8*, which further desaturates linoleic acid (18:2) to form α-linolenic acid (18:3). Besides these desaturations, *FAD5*, encoding a palmitoyl-monogalactosyldiacylglycerol delta(δ)-7 desaturase, affects the accumulation of 16:3 by catalyzing 16:0 MGDG to form 16:1 MGDG at position (Δ7) in leaves (Li-Beisson et al., 2013; Wang et al., 2015; Hernandez et al., 2016).

In the present study, we found unigenes corresponding to *SSI2, FAD2, FAD5, FAD7, FAD8*, and homologs of *Acyl-ACP thioesterase* that showed differential expression between seed and pericarp, which providing candidate genes for their difference in fatty acid compositions. The most surprizing results come from the *FAD2* isoforms. Although both seed and pericarp tissues have very high *FAD2* gene expression, which is consistent with their high C18:2 synthesis ability. The differences in the expression profiles of distinct of *FAD2* paralogous genes indicate that there may exist a tissue-specific transcriptional specialization mechanism to fine-tune *FAD2* transcription levels and therefore linoleic acid levels. Thus, subfunctionalization or neofunctionalization of *FAD2* genes through transcription diversification may lead to their different fatty acid metabolism during evolution. More importantly, it seems contradictory that the most highly expressed *FAD2* isoform c56614_g1 showed higher expression in pericarp with relatively lower C18:2 content, but not in seed. This result suggests that factors other than gene expression levels are also involved in the final C18:2 content. We suppose that: First, since many other fatty acid modification genes exhibits differential expression between seed and pericap, these enzymes may limit or compete with the *FAD2* synthesis ability for C18:2 in pericarp. For example, two different stearoyl-ACP desaturases were predominant in seed and pericarp respectively. Although their biochemical activity is not determined, it is possible that their product C18:1 (FAD2's substrate) may limit the activity potential of FAD2. Furthermore, the activities of each FAD2 isoform toward substrates are also unknown, which may result in difference in the final net C18:2 biosynthetic capabilities in the two tissues. Second, post-translational modifications including phosphorylation or ubiquitination degradation has been reported to down-regulate FAD2 activity (Tang et al., 2005). Interestingly, protein kinase activity pathway was over-represented (*p* = 1.58E-30) in pericarp upregulated genes through KEGG analysis. So, further work is of urgent need to directly determine the biochemical activity or post-translational modifications of FAD2 enzyme. Third, as the fatty acids are assembled in TAG finally, the major TAG assembly route will have selective to different kind of fatty acids. In *Arabidopsis, in vitro* enzyme activity analysis have demonstrated that different GPAT and LPAT genes, showed various activities to distinct acyl-CoA dependent on fatty acid length and unsaturation status (Kim et al., 2005; Yang et al., 2012). In correspondence with this point, we also found functional divergence of the members in GPAT and LPAT family between seed and pericarp through expression analysis (Table 6). These enzymes' substrate selective is an interesting question in the future.

**Figure 6:**
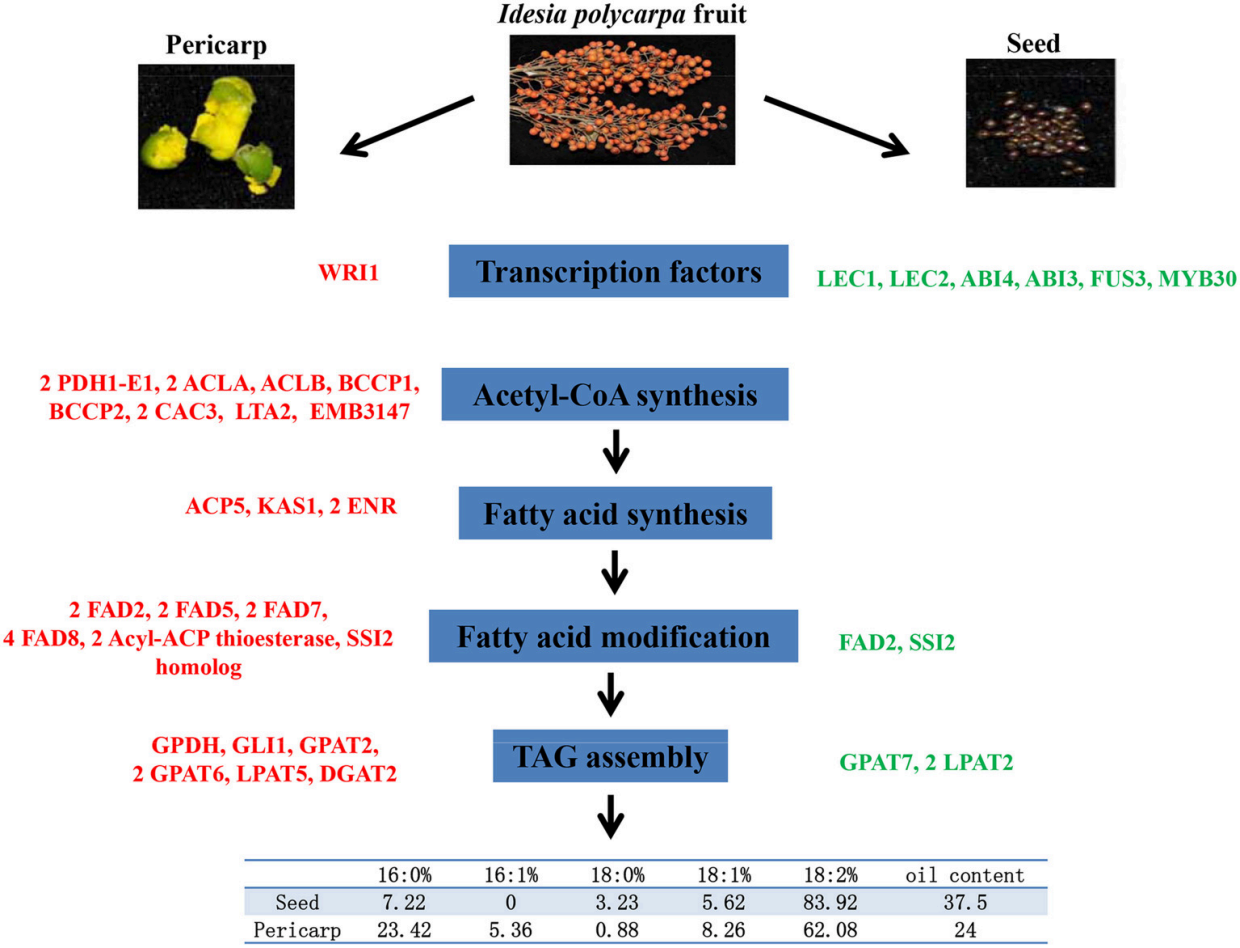
**Candidate genes for lipid synthesis**. Genes in red indicate higher expression in pericarps, while genes in green indicate higher expression in seeds.

Together, our findings from biochemical and transcriptomic comparison of seed and pericarp in *I. polycarpa* fruit lead to a proposed molecular basis for their difference in oil content and fatty acid compositions (Figure 6). Coordinated expression of Acetyl-CoA synthesis and fatty acid synthesis related genes were up-regulated by homolog of *WRI1* in pericap, resulting in improved lipid synthesis. Although such transcription patterns showed similarities to that in other oilseeds, important differences could be noted as it should be independent of the well-known seed maturation transcription factors (such as *LEC1, LEC2* etc.). An isoform of *FAD2* gene c56614_g1 expressed at significantly high level in both seed and pericarp, which may be responsible for their extraordinary content of linoleic acid. Furthermore, differential expression of 2 *FAD5* genes, could be involved in specific accumulation of palmitoleic acid in pericarp. Finally, complex expression or function diversification of fatty acid desaturase (*FAD2, SSI2* etc.,) and TAG assembly enzymes (GPAT and LPAT), may result in the considerable difference of fatty acid composition in seed and pericarp. Further investigations of the functions of these candidate genes by transgenic approaches in model organisms are areas for near future research. The results may help to manipulate the fatty acid composition and oil contents of oilseeds by means of genetic engineering.

## Author contributions

RL and SL designed the experiment; RL, XG, LL, and XL performed the experiments and analyzed the data; RL and SL wrote the manuscript; all authors reviewed and edited the manuscript.

### Conflict of interest statement

The authors declare that the research was conducted in the absence of any commercial or financial relationships that could be construed as a potential conflict of interest.

## Supplementary material

Figure S1 **Unigene distribution from the sequenced transcriptome**. The cDNA sequences were determined by Illumina HiSeq 2000 Sequencing System and *de novo* assembled using Trinity program.

Figure S2 **Species distribution of the top BLAST hits for reads in NR database**.

Figure S3 **GO classification of unigenes**. The y-axis on the top indicates the number of genes, and the y-axis on the bottom shows the percent of genes in a category.

Figure S4 **KOG classification of unigenes**. The y-axis on the top indicates the number of genes, and the y-axis on the bottom shows the percent of genes in a category.

Figure S5 **KOBAS analysis of differentially expressed genes**.

Table S1 **Unigenes functional annotation results**.

Table S2 **List of KEGG annotated unigenes**.

Table S3 **Top 50 expressed genes in seeds**.

Table S4 **Top 50 expressed genes in pericarps**.

Table S5 **Differentially expressed genes between seeds and pericarps**.

Table S6 **Lipid metabolism genes have *Arabidopsis* homologs**.

Table S7 **Differentially expressed lipid metabolism genes with *Arabidopsis* homologs**.

Table S8 **Primers used for qRT-PCR**.
