# Supplementary material for: De novo Assembly and Characterization of the Fruit Transcriptome of Idesia polycarpa Reveals Candidate Genes for Lipid Biosynthesis
Source: Front Plant Sci. 2016 Jun 7;7:801. doi: 10.3389/fpls.2016.00801 (PMC4896211; doi:10.3389/fpls.2016.00801)
Supplement: Figure S1 — Unigene distribution from the sequenced transcriptome. The cDNA sequences were determined by Illumina HiSeq 2000 Sequencing System and de novo assembled using Trinity program. [file Image1.PDF]

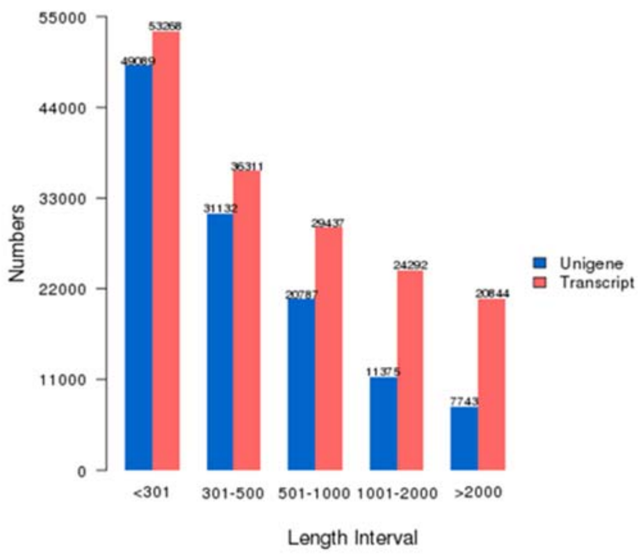


| Transcript length interval | 200-500bp | 500-1kbp | 1k-2kbp | >2kbp | Total |
|---|---|---|---|---|---|
| Number of transcripts | 89579 | 29437 | 24292 | 20844 | 164152 |
| Number of unigenes | 80221 | 20787 | 11375 | 7743 | 120126 |

| | Min Length | Mean Length | Median Length | Max Length | N50 | N90 | Total Nucleotides |
|---|---|---|---|---|---|---|---|
| Transcripts | 201 | 904 | 441 | 16722 | 1771 | 322 | 148411746 |
| Unigenes | 201 | 652 | 348 | 16722 | 1057 | 259 | 78357322 |
