# Supplementary figures and images for: De novo Assembly and Characterization of the Fruit Transcriptome of Idesia polycarpa Reveals Candidate Genes for Lipid Biosynthesis
Source: Front Plant Sci. 2016 Jun 7;7:801. doi: 10.3389/fpls.2016.00801 (PMC4896211; doi:10.3389/fpls.2016.00801)

### Species classification

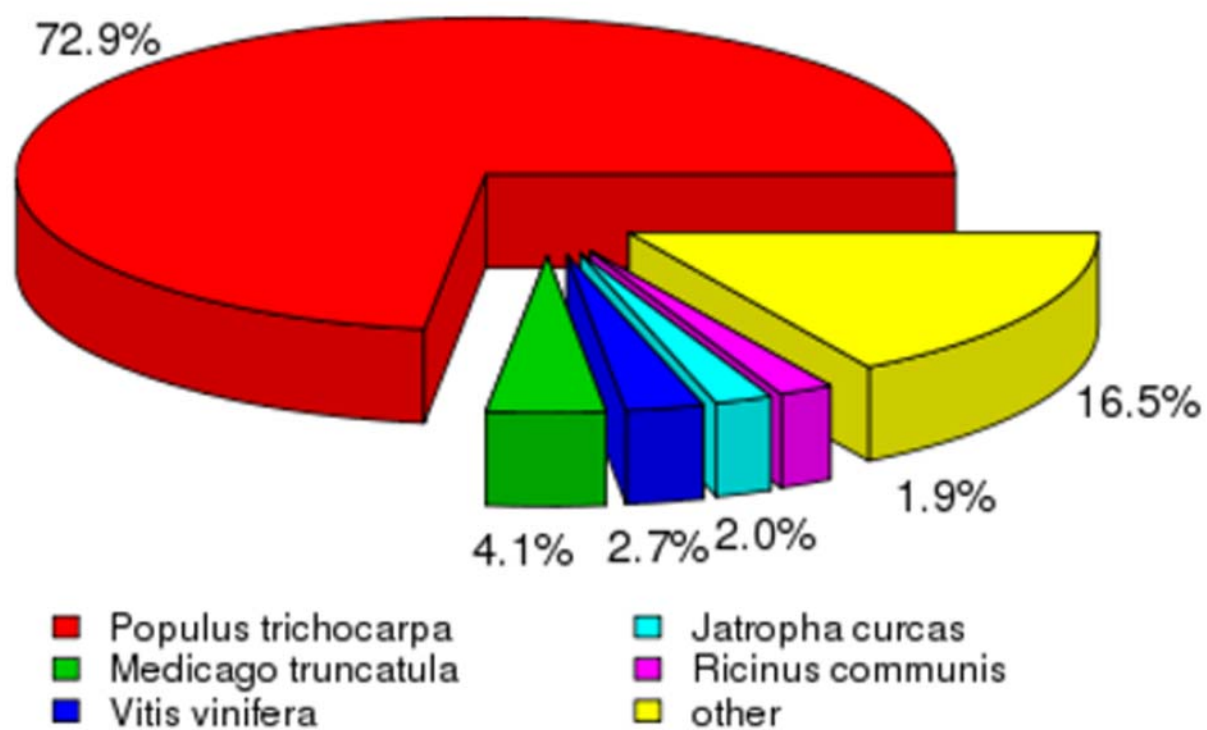

Supplement: Figure S2 — Species distribution of the top BLAST hits for reads in NR database. [file Image2.PDF]

# Statistics of Pathway Enrichment

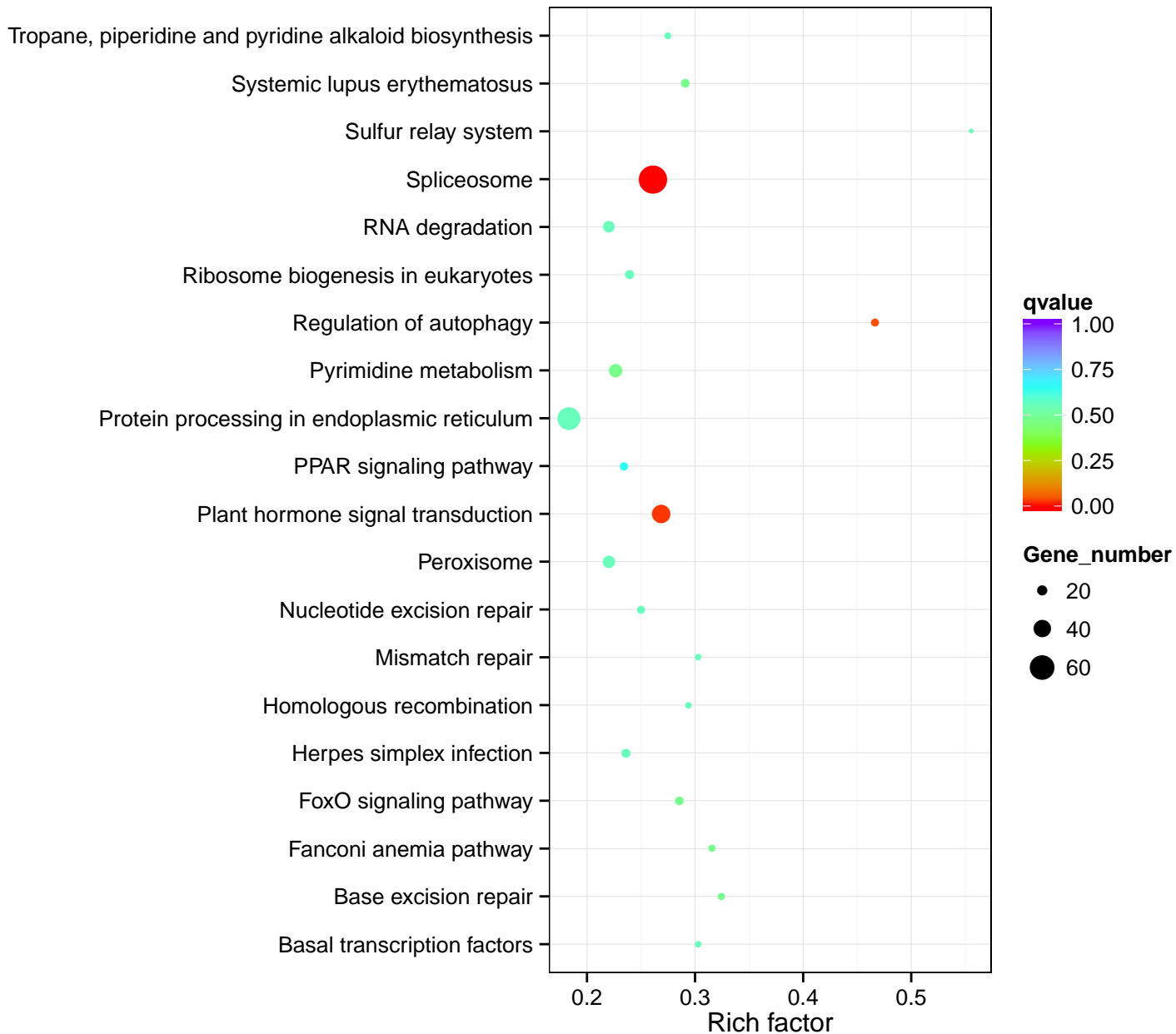

# Statistics of Pathway Enrichment

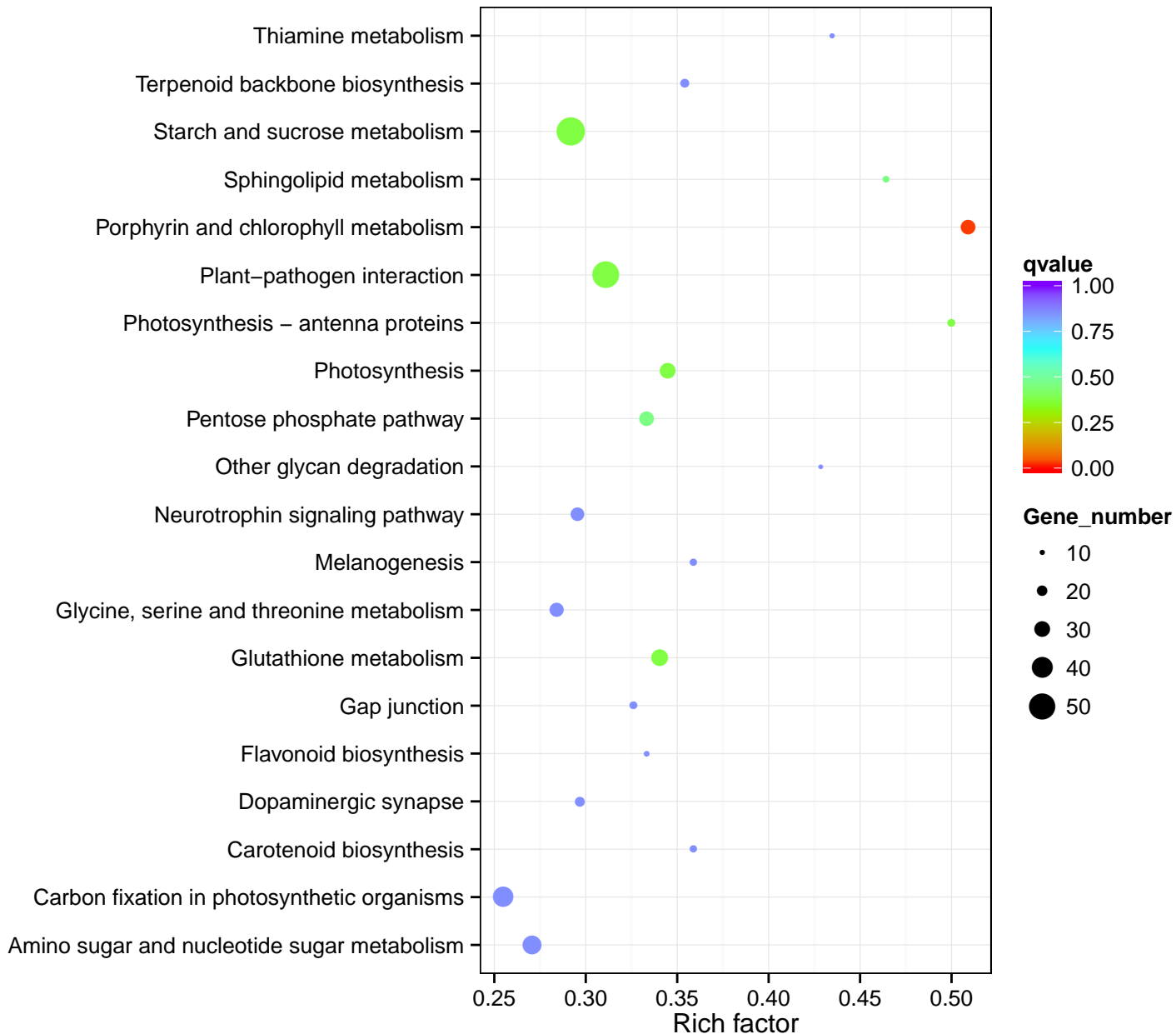

Supplement: Figure S5 — KOBAS analysis of differentially expressed genes. [file Image5.PDF]
