# Supplementary material for: De novo Assembly and Characterization of the Fruit Transcriptome of Idesia polycarpa Reveals Candidate Genes for Lipid Biosynthesis
Source: Front Plant Sci. 2016 Jun 7;7:801. doi: 10.3389/fpls.2016.00801 (PMC4896211; doi:10.3389/fpls.2016.00801)
Supplement: Figure S4 — KOG classification of unigenes. The y-axis on the top indicates the number of genes, and the y-axis on the bottom shows the percent of genes in a category. [file Image4.PDF]

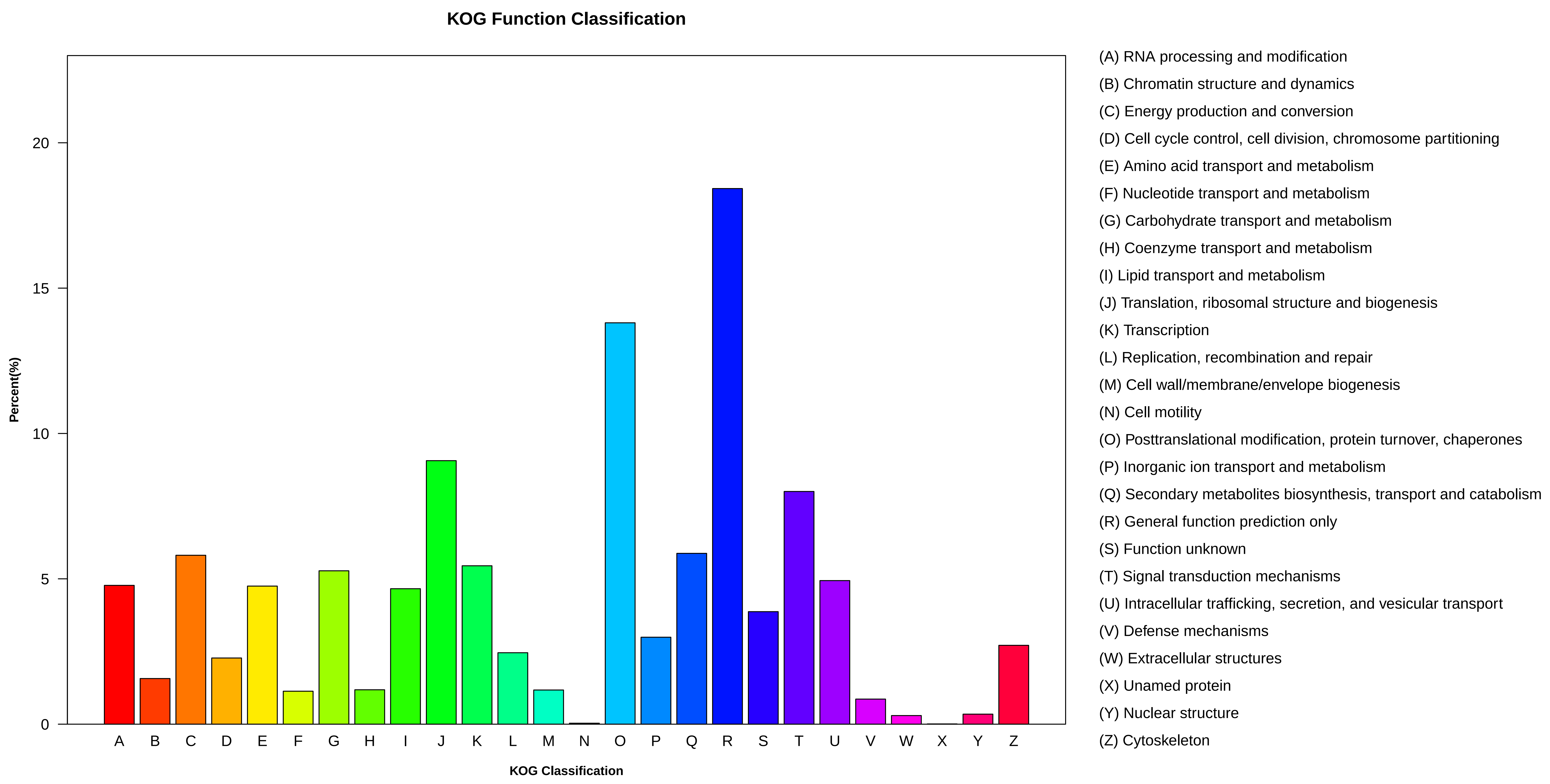
KOG Function Classification
Percent(%)
0
5
10
15
20
A B C D E F G H I J K L M N O P Q R S T U V W X Y Z
KOG Classification
(A) RNA processing and modification
(B) Chromatin structure and dynamics
(C) Energy production and conversion
(D) Cell cycle control, cell division, chromosome partitioning
(E) Amino acid transport and metabolism
(F) Nucleotide transport and metabolism
(G) Carbohydrate transport and metabolism
(H) Coenzyme transport and metabolism
(I) Lipid transport and metabolism
(J) Translation, ribosomal structure and biogenesis
(K) Transcription
(L) Replication, recombination and repair
(M) Cell wall/membrane/envelope biogenesis
(N) Cell motility
(O) Posttranslational modification, protein turnover, chaperones
(P) Inorganic ion transport and metabolism
(Q) Secondary metabolites biosynthesis, transport and catabolism
(R) General function prediction only
(S) Function unknown
(T) Signal transduction mechanisms
(U) Intracellular trafficking, secretion, and vesicular transport
(V) Defense mechanisms
(W) Extracellular structures
(X) Unamed protein
(Y) Nuclear structure
(Z) Cytoskeleton
